# Structural Dynamics of Temperature- and Pressure-Induced Phase Transitions in Hybrid Imidazolium Lead Chlorides

**DOI:** 10.1021/acs.inorgchem.6c00645

**Published:** 2026-06-15

**Authors:** Szymon Smółka, Dawid Drozdowski, Dagmara Stefańska, Anna Gągor, Adam Sieradzki, Katarzyna Fedoruk-Piskorska, Yuri da Silva Borges, Waldeci Paraguassu, Maciej Ptak

**Affiliations:** † Polish Academy of Sciences, 215275 Institute of Low Temperature and Structure Research, Okólna 2, 50-422 Wrocław, Poland; ‡ Faculty of Fundamental Problems of Technology, Wrocław University of Technology, Wybrzeze Wyspiańskiego 27, 50-370 Wrocław, Poland; § August Chelkowski Institute of Physics, University of Silesia in Katowice, 41-500 Chorzów, Poland; ∥ Department of Physics, Federal University of Pará, Belém 66075-110, PA, Brazil

## Abstract

We report the synthesis and comprehensive characterization of two previously unreported imidazolium lead chlorides, IM_2_Pb_3_Cl_8_·0.5H_2_O and IM_3_PbCl_5_, establishing chloride analogues as a platform to interrogate how inorganic-framework dimensionality and hydrogen-bond topology control structure, dynamics, and functional response in hybrid lead halides. Single-crystal X-ray diffraction reveals that IM_3_PbCl_5_ consists of one-dimensional corner-sharing [PbCl_5_]*_n_* ^3–^ chains, whereas IM_2_Pb_3_Cl_8_·0.5H_2_O adopts a three-dimensional porous framework constructed from edge- and face-sharing PbCl_8_ polyhedra hosting IM^+^ cations and water molecules. Variable-temperature calorimetry, diffraction, and Raman spectroscopy show that IM_3_PbCl_5_ undergoes a sequence of first-order phase transitions driven by progressive ordering of the imidazolium sublattice coupled to distortions of the inorganic chains and reorganization of N–H···Cl contacts; hydrostatic compression induces three additional transitions at elevated pressures. In contrast, IM_2_Pb_3_Cl_8_·0.5H_2_O shows no thermal anomalies in the investigated range and exhibits smaller pressure-induced shifts of selected phonon modes. Dielectric spectroscopy indicates thermally activated imidazolium reorientations and ionic transport at elevated temperatures, while optical measurements indicate wide band gaps and intense broadband emission in the spectral range from Vis to NIR.

## Graphical abstract

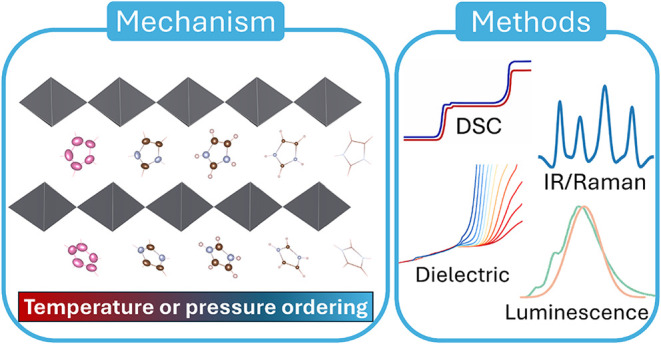

## Introduction

Hybrid organic–inorganic lead halides form a structurally diverse class of solids in which the connectivity and distortion of the inorganic framework, together with the arrangement of the organic component, can strongly influence lattice dynamics and optical response.^1,2^ This sensitivity is especially evident in low-dimensional architectures, where relatively small changes in Pb–X–Pb geometry or framework corrugation can substantially modify phonon behavior and photoluminescence. Temperature and hydrostatic pressure are particularly useful composition-preserving stimuli in such systems, as they can induce octahedral tilts, shorten Pb–X distances, alter symmetry, and reorganize phonon spectra, thereby driving phase transitions (PTs) and revealing the coupling between local structure and functional response.^3–12^ In low-dimensional lead-halide materials, large octahedral tilting and corrugation have been linked to broadband emission, whereas variable-temperature and high-pressure Raman and photoluminescence studies, supported by X-ray diffraction, further show that these responses are closely coupled to structural distortion, hydrogen-bond (HB) reorganization, and organic cation dynamics.^2,8,12–16^

Beyond local distortion, the topology of the inorganic sublattice is itself a key factor governing the properties of hybrid lead halides. Variations in dimensionality, from layered (2D) to chain-like (1D) and more weakly connected motifs, together with the way neighboring octahedra are linked, corner-, edge-, or face-sharing, determine the degree of electronic coupling within the inorganic framework.^17,18^ In lead iodide hybrids, a progression from corner- to edge- to face-sharing connectivity was shown to systematically widen the band gap,^18^ whereas studies of 1D compounds demonstrated that the topology of the chains and the Pb–I–Pb geometry influence the optical gap more strongly than the interchain separation.^19^ A broader experimental comparison across hybrid lead bromides, spanning 1D face-sharing to 3D corner-/edge-sharing frameworks, likewise confirmed that dimensionality and polyhedral linkage are accompanied by systematic changes in local distortion and band gap values.^20^

Chloride analogues are particularly informative in hybrid lead-halide comparisons because increasing Cl content typically contracts the lattice and shifts lattice modes to higher wavenumbers, consistent with stronger Pb–Cl interactions.^21^ Halide substitution can also alter inorganic connectivity and hydrogen-bond-mediated cation–framework coupling: in cyclic-ammonium lead halides, chloride and bromide members adopt different ribbon-, chain-, and layered motifs,^22^ and in 1D hybrid metal halides, the choice of halogen changes both chain packing and the asymmetric HB pattern around the inorganic framework.^23^ Consistent with this trend, spectroscopic analysis has shown stronger cation–halide interaction for chloride than for heavier halides.^24^

Within this broader family, compounds containing imidazolium cation (IM^+^) are of special interest because the ring is small, rigid, aromatic, and strongly anisotropic, which makes its orientational behavior and N–H···X interactions especially relevant for crystal packing and structural transformations. Only a few imidazolium lead halides have been reported, and, to our knowledge, none incorporate chloride.

Among bromides, IMPbBr_3_ adopts a distorted 4H hexagonal 3D framework with mixed face- and corner-sharing octahedra, whereas IM_2_PbBr_4_ and IM_3_PbBr_5_ form chain architectures, edge-shared double chains and corner-shared single chains, respectively. Temperature-dependent crystallography and lattice-dynamical studies have shown a rich sequence of PTs in these materials and linked them to cation ordering, octahedral distortions, and hydrogen-bond rearrangements.^25–27^ It has been demonstrated that IM_3_PbBr_5_ undergoes a sequence of transitions upon cooling, from the highly disordered *Cmmm* high-temperature (HT) phase to the partially disordered *P*1̅ phase at 395 K and finally to the ordered *P*1 low-temperature (LT) phase at 141 K.^26^ A polar order of IM^+^ cations in the LT phase results in the spontaneous polarization of 4.54 μC·cm^–2^.^28^ The LT phase is also formed above 0.33 GPa.^29^ Furthermore, IM_3_PbBr_5_ exhibits a band gap of 3.52 eV and a broadband excitonic yellow emission with a maximum at 574 nm.^26^

For iodides, IMPbI_3_ crystallizes in *P*6_3_/*m* as infinite face-sharing chains with staggered iodide arrangements, with the octahedral columns surrounded by six imidazolium ions and connected only by weak N–H···I interactions; pronounced disorder of the imidazolium sublattice couples to local iodide positional disorder within the columns.^1^ IM_2_Pb_2_I_7_ adopts a quasi-2D bilayer architecture in which a Pb_2_I_7_ slab is separated by a double layer of imidazolium cations.^30^ These bromide and iodide phases underscore the structural diversity enabled by IM^+^; chlorides, however, remain unexplored. Chlorides usually feature stronger Pb–Cl bonds, smaller lattice parameters, and higher characteristic phonon frequencies, as well as distinct N–H···Cl hydrogen bonding, all of which can reshape electron–phonon coupling and the propensity for self-trapping excitons (STE).^31–34^

Here, we report and characterize two novel imidazolium lead chloride crystals, IM_2_Pb_3_Cl_8_·0.5H_2_O and IM_3_PbCl_5_. The former has no known halide analogue, but IM_3_PbCl_5_ exhibits some similarities and differences in relation to IM_3_PbBr_5_,^26,29,35^ which are discussed in this paper. Temperature- and pressure-dependent Raman spectroscopy and temperature-dependent X-ray diffraction techniques are employed to track structure evolution, octahedral tilts, distortions, and hydrogen-bond reorganization under compression and changes in temperature. To gain further insight into the dynamical processes, the analysis is supported by thermal, dielectric, and AC conductivity measurements. In addition, linear and nonlinear optical properties were analyzed for both crystals.

## Experimental Section

### Materials and Synthesis

All reagents (PbCl_2_, ≥ 99%; imidazole, 99%; HCl, 37 wt % in H_2_O) were purchased from Sigma-Aldrich and used without further purification. Single crystals were grown through crystallization from acidic chloride media by slow evaporation at room temperature. For IM_2_Pb_3_Cl_8_·0.5H_2_O, a clear solution was prepared by dissolving imidazole and PbCl_2_ in concentrated HCl using an imidazole:PbCl_2_ molar ratio of 3:1. After stirring for a few minutes, the solution was left to crystallize at RT. After 1 week, small yellow cube-like monocrystals (1–2 mm) were harvested, and the mother liquid was allowed to evaporate further; after 5 weeks, a single crystal up to 1 cm was obtained (Figure S1b). For IM_3_PbCl_5_, the same procedure was followed with an imidazole:PbCl_2_ molar ratio of 5:1, affording needle-like colorless crystals within 1 week (Figure S1a). Crystals were harvested by decanting and air-dried at RT.

### Powder X-ray Diffraction

Powder XRD patterns were measured in the reflection mode on an X’Pert PRO X-ray diffraction system equipped with a PIXcel ultrafast line detector and Soller slits for Cu Kα_1_ radiation (λ = 1.54056 Å). The agreement between experimental powder XRD patterns and those simulated from single-crystal data confirms the phase purity of the samples (Figure S2).

### Single-Crystal X-ray Diffraction

Experiments were carried out using an Oxford Diffraction (OD) Xcalibur four-circle diffractometer equipped with the Atlas CCD detector, using graphite-monochromated Mo Kα_1,2_ radiation (λ = 0.7107 Å). Absorption corrections were applied using a multiscan method in CrysAlis PRO 1.171.42.93a (Rigaku OD, 2023). An empirical absorption based on spherical harmonics was implemented using the SCALE3 ABSPACK algorithm. Crystal structures were solved in Olex2 1.5 using the SHELXT program for structure solution and SHELXL for refinement.^36–38^ Hydrogen atoms were placed in calculated positions and refined as riding atoms. The main experimental and geometric parameters are summarized in Tables S1–S4, while HB parameters are listed in Tables S5–S6. Brief structural and refinement details are provided below.

IM_3_PbCl_5_ (**I**, 405 K): orthorhombic, *Cmmm*, *a* = 9.109(3) Å, *b* = 18.200(3) Å, *c* = 5.898(5) Å, *V* = 977.7(6) Å^3^, *Z* = 2, *R*_1_ = 0.04, *w*R**_2_ = 0.08, *S* = 1.01; (**II**, 295 K): monoclinic, *P*2/*c*, *a* = 5.812(3) Å, *b* = 9.148(3) Å, *c* = 17.736(5) Å, β = 91.91(3)°, *V* = 942.6(6) Å^3^, *Z* = 2, *R*_1_ = 0.02, *w*R**_2_ = 0.05, *S* = 1.20; (**III**, 240 K): monoclinic, *P*2_1_/*c*, *a* = 5.775(3) Å, *b* = 17.702(5) Å, *c* = 18.263(5) Å, β = 90.07(3)°, *V* = 1867.0(12) Å^3^, *Z* = 4, *R*_1_ = 0.03, *w*R**_2_ = 0.06, *S* = 1.02; (**IV**, 120 K): monoclinic, *P*2_1_/*c*, *a* = 5.747(3) Å, *b* = 17.567(5) Å, *c* = 18.213(5) Å, β = 90.22(3)°, *V* = 1838.9(12) Å^3^, *Z* = 4, *R*_1_ = 0.02, *w*R**_2_ = 0.03, *S* = 1.05.

IM_2_Pb_3_Cl_8_·0.5H_2_O (100 K): monoclinic, *C*2/*c*, *a* = 21.119(6) Å, *b* = 10.687(4) Å, *c* = 35.625(9) Å, β = 99.41(3)°, *V* = 7932(4) Å^3^, *Z* = 16, *R*_1_ = 0.03, *w*R**_2_ = 0.08, *S* = 1.15.

### Differential Scanning Calorimetry (DSC)

DSC measurements were performed using a Mettler Toledo DSC-3 calorimeter with a resolution of 0.4 μW. Nitrogen was used as a cooling gas to maintain thermal stability during the measurements. The heating and cooling rates of 5 K·min^–1^ were applied. The sample masses were 12.2 mg for IM_3_PbCl_5_ and 24.4 mg for IM_2_Pb_3_Cl_8_·0.5H_2_O. The excess heat capacity associated with the phase transitions was determined by subtracting a baseline representing the lattice contribution. The baseline was constructed by fitting the DSC signal in temperature regions outside the transitions and extrapolating it through the transition range. The difference between the measured heat flow and this baseline was then used to evaluate the anomalous contribution.

### Broadband Dielectric Spectroscopy (BDS)

Dielectric measurements were carried out using a broadband impedance analyzer, Novocontrol α-A, over a frequency range of 1 Hz to 1 MHz. For all of the studied compounds, measurements were conducted on single crystals. To ensure good electrical contact, the crystal surfaces were coated with silver paste. Temperature was controlled by using a Novocontrol Quattro system equipped with a nitrogen cryostat, providing stability to better than 0.1 K.

### Infrared Measurements (IR)

RT mid-IR transmittance spectra (4000–400 cm^–1^, 2 cm^–1^ resolution) were recorded using a Thermo Scientific Nicolet iS50 FT-IR spectrometer. Samples were prepared as Apiezon N mulls on ZnSe pellets.

Temperature-dependent IR measurements for IM_2_Pb_3_Cl_8_·0.5H_2_O and IM_3_PbCl_5_ were carried out from 7 to 300 K using a Nicolet iS50 IR spectrometer combined with cryostat CS202AE-DMX-1AL (Advanced Research System). The cryostat was equipped with thallium bromide-iodide windows. Since the chlorides react with KBr, temperature-dependent measurements in the Apiezon N mulls were performed only at RT.

### Raman Measurements

RT Raman spectra of polycrystalline samples (3500–50 cm^–1^, 2 cm^–1^ resolution) were measured using a Bruker MultiRAM FT-Raman spectrometer equipped with a 1064 nm Nd:YAG laser.

Temperature-dependent Raman spectra of IM_2_Pb_3_Cl_8_·0.5H_2_O and IM_3_PbCl_5_ single crystals (3600–50 cm^–1^) were measured using a Renishaw inVia system equipped with a Leica DM2500 confocal microscope and 488 nm excitation laser line. The temperature, with stability up to 0.1 K, was controlled using a Linkam cryostat cell THMS600 stage equipped with quartz windows. Measurements above 300 K were not performed due to unexpected sample luminescence.

High-pressure Raman measurements were carried out using a Labram Evolution spectrometer (Horiba) equipped with a microscope. A 514.5 nm laser line was used as the excitation source, and the spectral resolution was set to 2 cm^–1^. High-pressure conditions were generated using a Diacell μScopeDAC-RT­(G) diamond anvil cell (Almax easyLab) equipped with diamonds with 0.4 mm culets. The sample was placed in a 120 μm diameter hole drilled in a 200 μm thick stainless-steel gasket using an electric discharge machine (Almax easyLab). Nujol (mineral oil) served as the pressure-transmitting medium. The pressure was evaluated from the shifts of the ruby R_1_ and R_2_ fluorescence lines.

### Emission and Absorption Properties

RT absorption spectra of the powdered samples were measured using a Varian Cary 5E UV–Vis–near-IR (NIR) spectrophotometer. Emission spectra at various temperatures under 266 nm excitation from a diode laser were measured with the Hamamatsu photonic multichannel analyzer PMA-12 equipped with a BT-CCD linear image sensor. The temperature of the single-crystal sample was controlled using a Linkam THMS600 heating/freezing stage.

## Results and Discussion

### Thermal Properties

The DSC thermogram of IM_3_PbCl_5_ reveals three reversible PTs occurring at *T*_3_ = 233.1 K (222.5 K), *T*_2_ = 250.7 K (245.1 K), and *T*_1_ = 399.2 K (396.4 K) during the heating (cooling) cycle (Figure 1). These transitions are characterized by sharp peaks in the DSC signal. The observed thermal hysteresis between the heating and cooling branches for each transition, combined with the abrupt changes in entropy, clearly indicates that all three PTs are of first-order character. To quantify the associated entropy changes (Δ*S*), the specific heat contribution was estimated and subtracted from the total DSC signal to isolate the anomalous component corresponding to latent heat. Integration of this anomalous heat flow over each transition range yielded total entropy changes of 18.0 J·mol^–1^·K^–1^ upon heating and 20.7 J·mol^–1^·K^–1^ upon cooling (see inset of Figure 1). The minor discrepancy between the heating and cooling values likely arises from uncertainties in defining the baseline for latent heat estimation and slight differences in transition kinetics. Notably, the entropy change associated with the PT at *T*_2_ is relatively small, suggesting that this transformation may be displacive in nature or with a weak contribution of ordering, involving only subtle changes in atomic positions without a significant change in crystal symmetry. In contrast, the substantially larger entropy changes observed at *T*_1_ and *T*_3_ imply significant structural reorganizations, indicative of PTs involving lattice symmetry breaking or more extensive atomic rearrangements. For IM_3_PbBr_5_, the observed DSC peak at *T*_1_ at 400/395 K has had a similar thermal hysteresis and magnitude (Δ*S* = 20.4 J·mol^–1^·K^–1^) and resulted from the partial ordering of IM^+^ cations. In contrast to IM_3_PbCl_5_, there was only one weaker PT at *T*_2_ = 143/141 K (5.3 J·mol^–1^·K^–1^) associated with a full IM^+^ ordering,^26^ suggesting differences in the ordering mechanisms.

**Figure 1:**
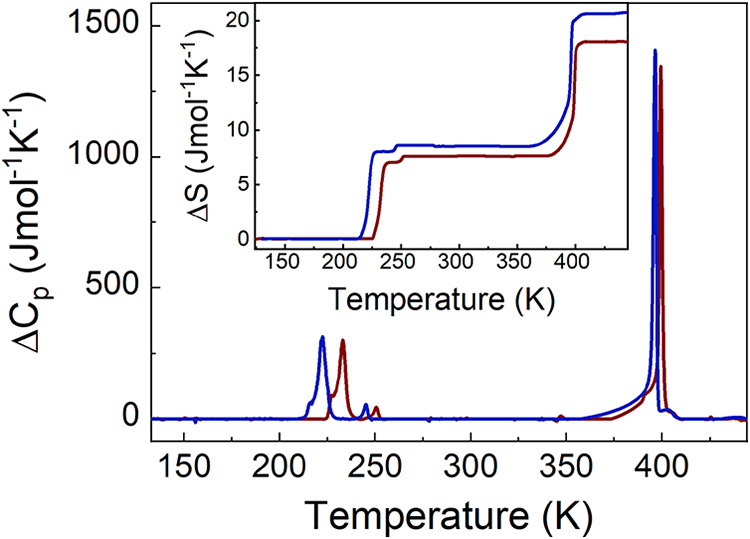
Temperature dependence of heat capacity anomaly (Δ*C*_p_) of IM_3_PbCl_5_ measured during cooling (blue) and heating (red) cycles. The inset shows the changes in entropy (ΔS) as a function of temperature.

DSC thermogram of IM_2_Pb_3_Cl_8_·0.5H_2_O shows no thermal anomalies within the investigated temperature range, indicating the absence of detectable phase transitions under the measured conditions (Figure S3).

### Single-Crystal X-ray Diffraction

IM_3_PbCl_5_ represents the 1D subclass of hybrid organic–inorganic lead halides. The inorganic structure consists of [PbCl_5_]*_n_* ^3–^ chains arising from the corner-sharing PbCl_6_ octahedra. Similar arrangements have already been reported for the IM_3_PbBr_5_ analogue,^26^ as well as for several hybrid lead chlorides.^23,39,40^

The HT phase (**I**, orthorhombic, *Cmmm*) is depicted in Figure 2a. The characteristic feature of this phase is a heavy disorder of all IM^+^ moieties, similarly to the isostructural HT analogue of IM_3_PbBr_5_.^26^ Upon cooling, a PT occurs and the symmetry is reduced to monoclinic, *P*2/*c* (phase **II**). The symmetry reduction is associated with a site-selective ordering/disordering of the organic cation, as shown in Figure 2b. IM^+^ that stack along the *b*-direction are ordered and anchored to PbCl_6_ octahedra via N–H···Cl HBs. In contrast, IM^+^ cations positioned along the polymeric [PbCl_5_]*_n_* ^3–^ chains—extending along the *a*-axis—exhibit in-plane disorder, as evidenced by split atomic positions in the diffraction data. The ordered IM^+^ units bridge two neighboring [PbCl_5_]*_n_* ^3–^ chains along the *c*-direction via bifurcated N–H···Cl HBs, additionally donating protons to two Cl^–^ acceptors from adjacent in-chain octahedra.

**Figure 2:**
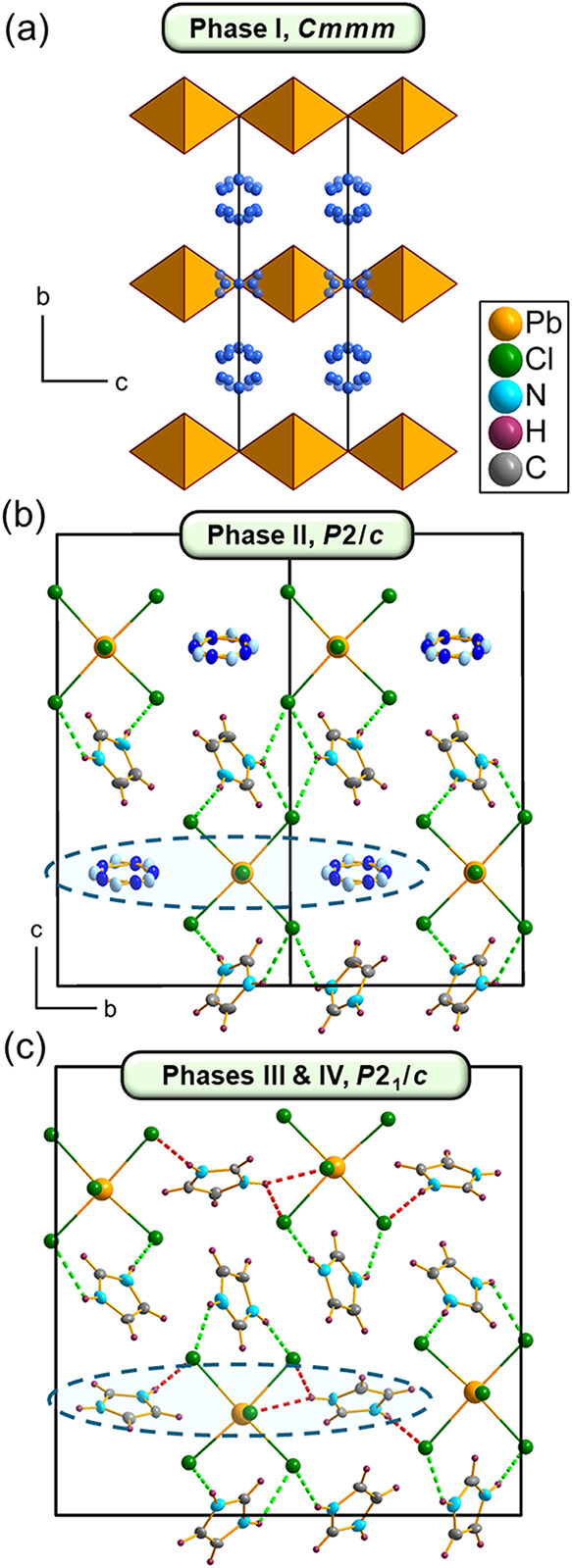
Crystal structure of IM_3_PbCl_5_ in (a) HT phase **I** (orthorhombic, *Cmmm*), (b) RT phase **II** (monoclinic, *P*2/*c*), and (c) LT phases **III** and **IV** (monoclinic, *P*2_1_/*c*). Ovals in (b) and (c) highlight the change in IM^+^ dynamics upon the **II** → **III** PT. Dashed lines represent N–H···Cl HBs. Interactions associated with IM^+^ ordering in **III** are marked in red.

Below 251 K, further ordering of IM^+^ cations triggers a reorganization of the HB network, which displaces the IM^+^ cation arrangement and leads to a PT into another monoclinic structure, described by the *P*2_1_/*c* space group (phase **III**). This transition (**II** → **III**) involves loss of translational symmetry (new translation vector *c*′ = 2*b*), resulting in a transformed unit cell described by the new metric: [100 001 020]. The crystal structure of phase **III** is presented in Figure 2c. In phase **III**, the ordering of IM^+^ cations is driven by the formation of new N–H···Cl HBs (highlighted as red dashed lines in Figure 2c), which now link adjacent [PbCl_5_]*_n_* ^3–^ chains in (*a*, *b*) planes. The IM^+^ cations positioned along [001] exhibit the shift of the geometric centers from the (002) plane. This shift results in reorganization of the HB network: the intrachain HBs observed in phase **II** are disrupted, and each IM^+^ cation now interacts exclusively with a single chain.

The last PT is isostructural and leads to phase **IV** (*P*2_1_/*c*), with the monoclinic β angle moderately increased—from 90.07(3)° to 90.22(3)°. The **III** → **IV** transition is likely to be caused by another reorientation of the IM^+^ cations, being subtle enough not to affect the symmetry of the crystal structure. A similar phenomenon was observed in the IMMHyPbX_4_ (MHy^+^ = methylhydrazinium; X = Br^–^, Cl^–^) 2D hybrid halides, where the gradual freezing of MHy^+^ dynamics occurred within a single phase and led to a dipolar relaxation process.^13^ The observed structural behavior also indicates that the relationship between crystallographic symmetry changes and DSC signal intensity (Figure 1) is not straightforward. In the studied compound, symmetry lowering occurs already during the **II** → **III** PT, whereas the larger calorimetric effect is associated with the subsequent ordering and freezing of IM^+^ cation dynamics during the **III** → **IV** transition.

The changes in symmetry and hydrogen bonding geometry also affect the inorganic framework. In phase **I**, the symmetry-independent inorganic unit consists of PbCl_2_ groups forming PbCl_6_ octahedra with *D*_2h_ point symmetry (Figure S4). In phase **II**, these are replaced by symmetry-independent PbCl_3_ units with *C*_2_ point symmetry, followed by PbCl_5_ units with *C*_1_ point symmetry in phases **III** and **IV**, respectively, indicating increased distortion in the lead coordination environment. Expressing this via octahedral distortion parameters, the bond length distortion increases modestly from 0.008 to 0.016, while the bond angle variance increases significantly, from 0.9 deg^2^ in phase **I** to 19.2 deg^2^ in phase **IV**, highlighting a notable angular deformation of the octahedra. Although the HT phase of IM_3_PbBr_5_ exhibits the same disorder pattern and the LT phase adopts triclinic symmetry, the corresponding PbBr_6_ distortions are smaller, increasing from 0.001 and 0.4 deg^2^ (HT) to 0.001 and 10.7 deg^2^ (LT).^26^

IM_2_Pb_3_Cl_8_·0.5H_2_O crystallizes in a monoclinic *C*2/*c* space group with a relatively large unit cell volume of 7932(4) Å^3^ at 100 K and *Z* = 16. The asymmetric unit (Figure S5) comprises six Pb^2+^ cations, 16 Cl^–^ anions, four IM^+^ cations, and one water molecule. The crystal structure is presented in Figure 3. The inorganic framework consists of distorted PbCl_8_ coordination polyhedra that share edges and faces, forming a 3D porous network with IM^+^ cations and water molecules located within the pores. Three of the IM^+^ cations are fully ordered and participate in N–H···Cl HB interactions with the inorganic framework. The fourth IM^+^ cation is disordered. At 295 K, it exhibits in-plane librations, while at 100 K, the atoms of its C–C chain are split over two independent sites, each with an occupancy factor of 0.5 (see Figure S5). This disordered cation additionally forms N–H···O HBs with the water molecule.

**Figure 3:**
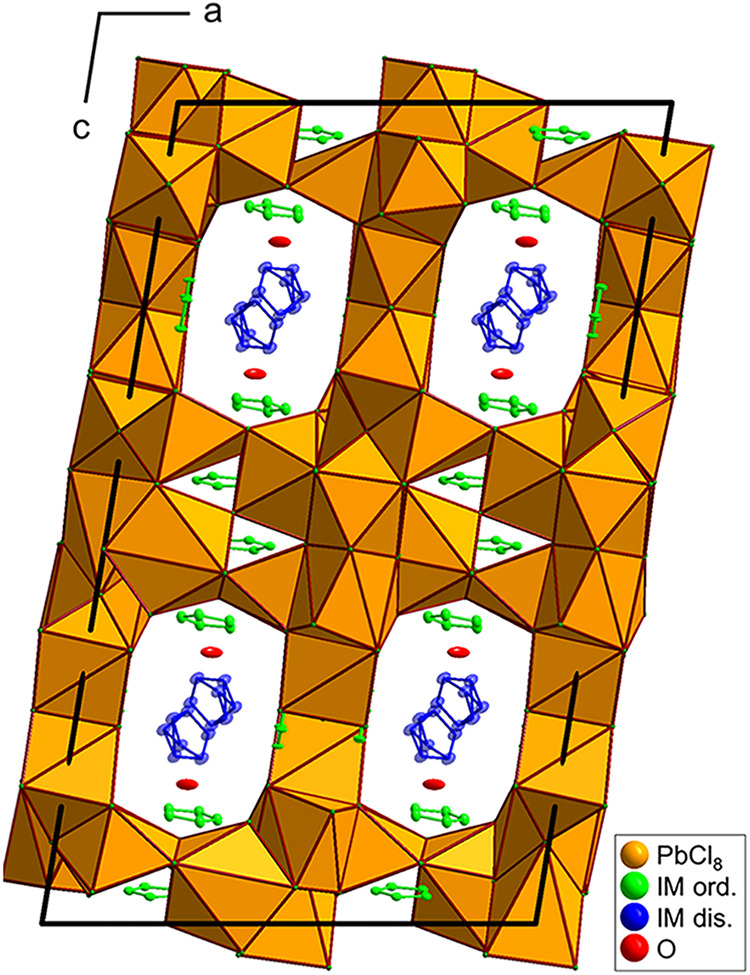
Crystal structure of IM_2_Pb_3_Cl_8_·0.5H_2_O in the monoclinic *C*2/*c* space group at 100 K. Ordered and disordered IM^+^ cations are marked with green and blue, respectively. H atoms are omitted for clarity.

### Dielectric Analysis

BDS was employed to investigate dipolar relaxation associated with the reorientational dynamics of molecular dipoles, as well as the electrical conductivity arising from the translational motion of charge carriers, including ions and electrons. The temperature dependence of the real part of dielectric permittivity (ε′) and dielectric loss (ε″) for IM_3_PbCl_5_ and IM_2_Pb_3_Cl_8_·0.5H_2_O is shown in Figure 4. For IM_3_PbCl_5_, subtle anomalies are observed near the structural phase transitions from phase **IV** to phase **III** and from phase **III** to phase **II** (Figure 4a), indicating changes in dipolar dynamics linked to structural changes. Furthermore, above approximately 250 K, frequency dispersion of ε* appears, indicating enhanced thermally activated charge transport. Such behavior is commonly observed in hybrid halide systems,^41–44^ although contributions from electrode polarization, trapped charges, and extended defects cannot be fully excluded. In contrast, IM_2_Pb_3_Cl_8_·0.5H_2_O exhibits relatively stable ε*′* at lower temperatures, followed by a sharp increase above 325 K, accompanied by pronounced frequency dispersion comparable to that of IM_3_PbCl_5_ (Figure 4b). This behavior reflects a similar onset of thermally activated charge transport.

**Figure 4:**
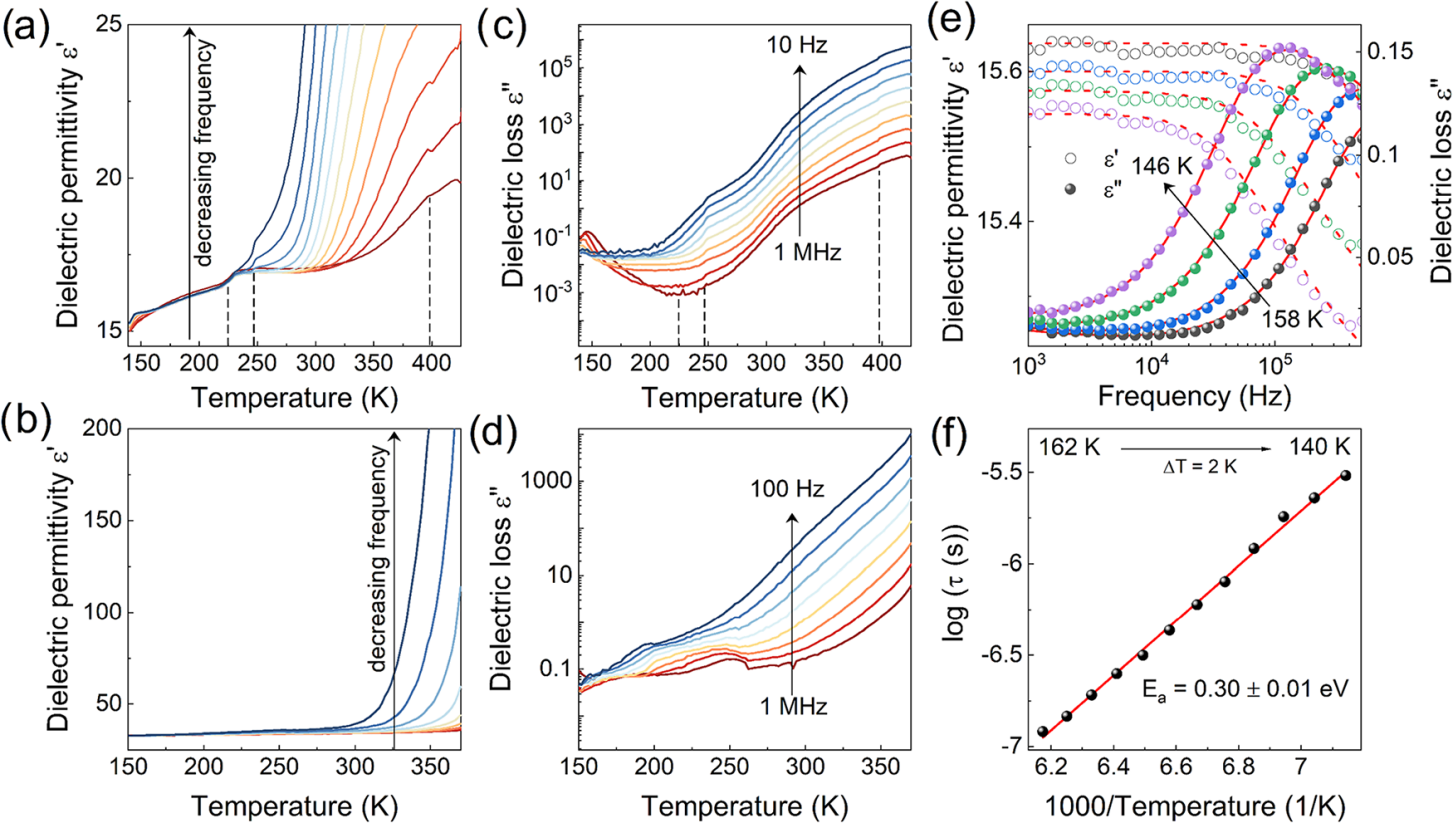
Temperature dependence of the dielectric properties for IM_3_PbCl_5_ and IM_2_Pb_3_Cl_8_·0.5H_2_O. The real part of the dielectric permittivity (ε′) is shown in (a) and (b), and the dielectric loss (ε″) in (c) and (d), respectively. Dashed vertical lines mark the structural phase transition temperatures. In (e), the frequency-dependent complex permittivity (ε*) of IM_3_PbCl_5_ is fitted using the Havriliak–Negami function, illustrating dipolar relaxation behavior. Plot (f) presents the logarithm of the relaxation time (log τ) as a function of inverse temperature (1000/*T*), with the data well described by the Arrhenius relation, confirming thermally activated dipole dynamics.

At low temperatures, the dielectric loss spectra of IM_3_PbCl_5_ reveal a distinct bell-shaped peak characteristic of dipolar relaxation processes (Figure 4c). In contrast, IM_2_Pb_3_Cl_8_·0.5H_2_O exhibits no such feature under comparable conditions (Figure 4d), suggesting the absence of significant structural dynamics. This dipolar relaxation behavior in IM_3_PbCl_5_ is further corroborated by its frequency-dependent complex permittivity, which shows characteristic dispersion consistent with thermally activated dipole dynamics (Figure 4e). To quantitatively describe this relaxation, the Havriliak–Negami function was employed, as it effectively captures the asymmetric and broadened relaxation peaks arising from a distribution of dipole relaxation times typical in disordered hybrid materials. The extracted relaxation times (τ) display Arrhenius-type temperature dependence over the measured range. Figure 4f was modeled by

$$\tau =\tau_{0}e^{E_{a}/k_{B}T}$$

where τ_0_ is the relaxation time extrapolated to the high-temperature limit, *k*_B_ is Boltzmann’s constant, and *E*_a_ denotes the activation energy. The estimated *E*_a_ of 0.30 eV corresponds well with the energy barrier for thermally activated reorientational motions of IM^+^ cations within the crystal structure, consistent with previous reports on bromide analogue (IM_3_PbBr_5_), which shows similar relaxation processes with activation energies of 0.30 and 0.10 eV.^13,26^

At low frequencies, the dielectric loss (ε″) exhibits an approximately inverse frequency dependence (ε″ ∝ 1/ω), corresponding to a slope close to −1 in the log–log representation. This behavior is characteristic of a dc conductivity contribution, indicating that charge transport significantly contributes to the dielectric response in this regime. To further analyze the high-temperature transport behavior, the electric modulus formalism was employed. The imaginary part of the electric modulus (*M*″) exhibits well-defined, bell-shaped frequency-dependent peaks for both materials (Figures S6a and S7a), indicative of a thermally activated relaxation process associated with charge transport.

These peaks were fitted using the Havriliak–Negami function:

$$M^{*}\left(\omega \right)=M_{\infty }+\frac{M_{0}-M_{\infty }}{{\left[1+{\left(i\omega \tau \right)}^{1-\alpha }\right]}^{\beta }}$$

where τ is the characteristic relaxation time, while α and β describe the symmetric and asymmetric broadening of the relaxation peak, respectively. The extracted relaxation times follow Arrhenius-type temperature dependence, as discussed above. The activation energies obtained from the modulus analysis are 0.54 ± 0.03 eV (phase **I**) and 0.79 ± 0.02 eV (phase **II**) for IM_3_PbCl_5_, indicating that structural phase transitions influence the transport process (Figure S6b). For IM_2_Pb_3_Cl_8_·0.5H2O, an activation energy of 0.64 eV was determined, suggesting a comparable thermally activated mechanism (Figure S7b). The presence of well-defined *M*″ peaks together with the consistent Arrhenius behavior of the relaxation times indicates that the observed response is governed by a dominant thermally activated charge-transport process, although contributions from extrinsic effects cannot be fully excluded.

### Phonon Studies

#### IM_3_PbCl_5_

Temperature-dependent IR and Raman spectra measured from 80 to 300 K of IM_3_PbCl_5_ (Figure 5a,b) show two low-temperature PTs that coincide with the DSC anomalies shown in Figure 1. Raman spectroscopy resolves the **III** → **II** transition at approximately 230–240 K and the **IV** → **III** PT at approximately 210–220 K, whereas IR spectroscopy effectively registers a single change spanning **IV** → **II**, as the intermediate state produces only weak spectral signatures.

**Figure 5:**
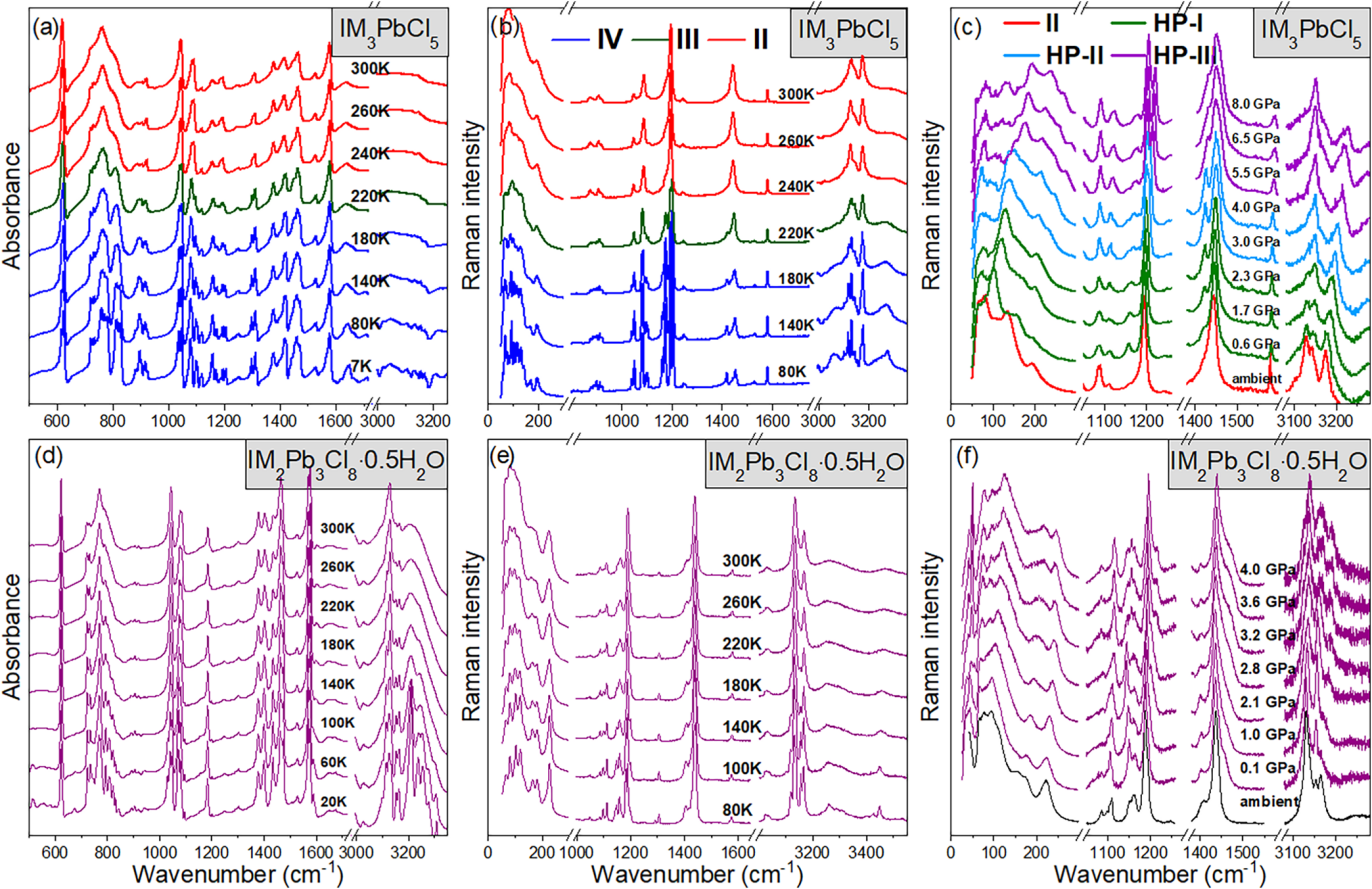
Temperature- and pressure-dependent vibrational spectra of imidazolium lead chlorides. Panels (a–c) correspond to IM_3_PbCl_5_, while panels (d–f) correspond to IM_2_Pb_3_Cl_8_·0.5H_2_O. In (a) and (d), infrared spectra are shown as a function of temperature; in (b) and (e), Raman spectra are shown as a function of temperature; and in (c) and (f), Raman spectra are shown as a function of pressure. For the pressure-dependent Raman measurements, traces are partitioned into segments separated by axis breaks and each segment is independently normalized. Curve labels indicate the measurement temperature or pressure, and colors denote the distinct phases observed.

At 80 K, both the IR and Raman bands are very narrow and split, confirming that phase **IV** is fully ordered. Narrow bands above 3100 cm^–1^, assigned to νNH and νNH^+^, indicate that the network of HBs is well-defined and strong enough to immobilize all IM^+^ cations. Upon heating to *T*_3_–*T*_2_, all IR bands gradually broaden, and this occurs more quickly for bands originating from vibrations of nitrogen atoms, suggesting a continuous increase in the cation librational freedom.

Figure 6a shows that, apart from the broadening of Raman and IR bands, the **IV** → **III** PT introduces modest changes. These changes primarily affect the low-wavenumber region probing the [PbCl_5_]*_n_* ^3–^ framework and selected HB-sensitive bands, with other spectral regions remaining essentially unchanged at this step up to *T*_3_. This behavior is consistent with the isostructural character of this PT. A moderate rise in entropy at *T*_3_ (**IV** → **III**) upon heating is most likely due to partial weakening in the HB network, accompanied by an increase in the thermal motion of atoms, as well as slight reorientations and changes in the PbCl_6_ deformations, as indicated by diffraction studies.

**Figure 6:**
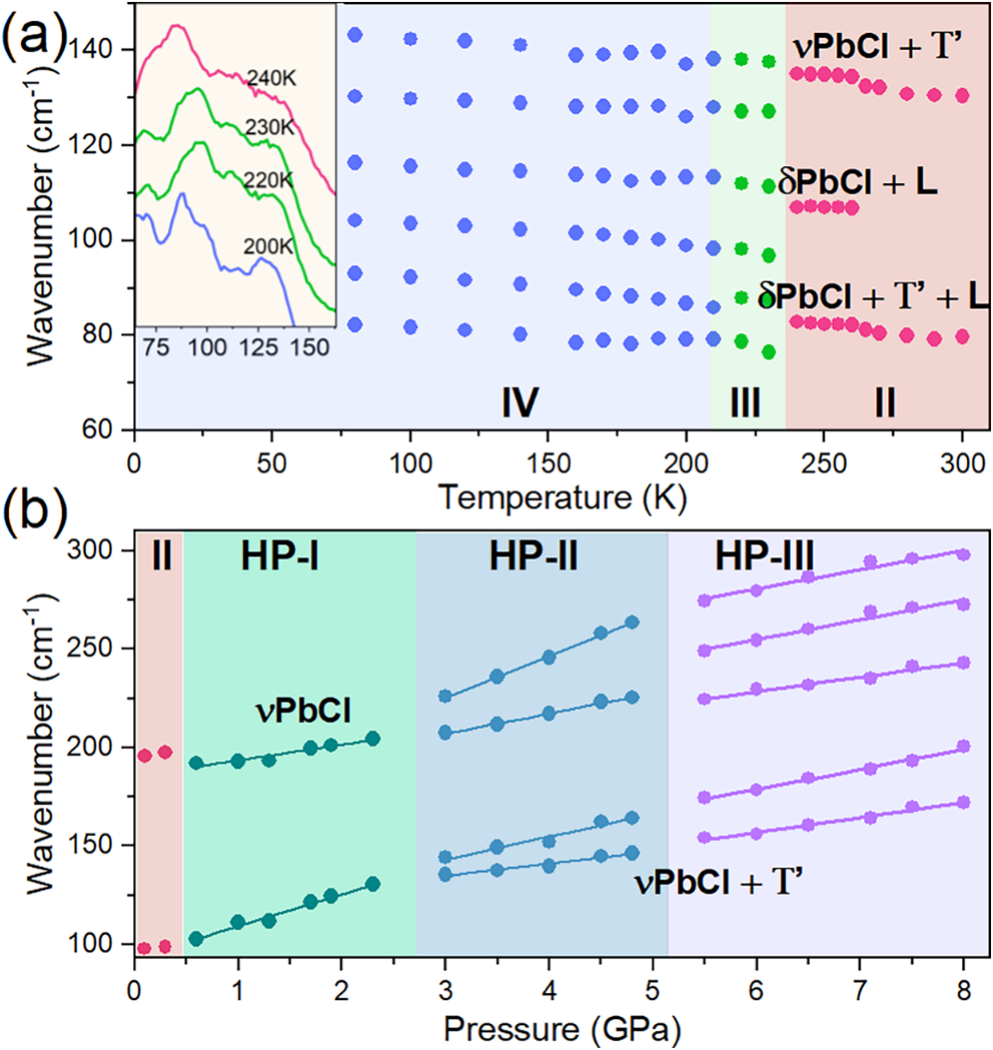
Evolution of positions of selected (a) temperature- and (b) pressure-dependent Raman bands of IM_3_PbCl_5_. Colors of full circles and rectangles fill correspond to different temperature phases and pressure phases. Each of the presented modes has labels with assignments: ν, stretching; δ, in-plane bending; T′, translation; and L, libration.

Entering phase **II** at *T*_2_ upon further heating, by contrast, reflects a diminished diversity of local environments: the PbCl_6_ low-wavenumber Raman bands and the IM^+^ ring-bending and stretching modes in the 600–950, 1000–1220, and 3000–3300 cm^–1^ ranges merge and simplify (assignments in Table S7 and band-position fits in Figures S8–S10), indicating the subtle change in monoclinic symmetry. IR spectra show a pronounced broadening and merging of bands in phase **II**, indicating dynamical reorientations of IM^+^ cations rather than static positional disorder. This interpretation is supported by Raman spectra, which exhibit broadening of bands associated with ring vibrations. The pronounced broadening and softening of bands in the low-wavenumber Raman spectra suggest stronger anharmonicity and a change in the coupling between IM^+^ and the inorganic sublattice.

These observations support a sequence in which **III** → **II** is related to the onset of the in-plane disorder of IM^+^ cations, while **IV** → **III** is associated with the unlocking of librational motions by weakening of the imidazolium–chlorides interactions. This interpretation reconciles the vibrational data with the DSC results, namely, a small increase in Δ*S* for the **III** → **II** transition, attributable to the onset of weak in-plane configurational reorientations, and a larger entropy change for the **IV** → **III** PT, which is consistent with the activation of enhanced librational mobility and the associated structural changes.

A direct analogy emerges when IM_3_PbCl_5_ is compared with its bromide counterpart. In IM_3_PbBr_5_, the HT phase **I** (*Cmmm*) is fully disordered, the RT phase **II** (*P*1̅) is partially ordered, exhibiting in-plane rotations of IM^+^, and the LT phase **III** (*P*1) is fully ordered.^29^ The chloride sequence aligns closely with this scheme: phases **I** and **II** correspond to the fully disordered and partially ordered regimes established for the bromide, while chloride phase **IV** corresponds spectroscopically to the fully ordered bromide phase **III**, including the characteristic band splitting. It is worth noting, however, that the corresponding transition in IM_3_PbCl_5_ is associated with a smaller entropy change than in IM_3_PbBr_5_.^29^ Furthermore, stronger HB interactions in IM_3_PbCl_5_ favor a stepwise unlocking of IM^+^ dynamics and result in an additional isostructural PT. This structural arrangement stabilizes a monoclinic symmetry of the LT phase, in contrast to IM_3_PbBr_5_, which adopts a triclinic LT structure.

To examine whether a related low-symmetry ordered state can be accessed by compression, we performed pressure-dependent Raman measurements up to 8 GPa (Figure 5c). Based on the mode evolution and individual peak tracking, the spectra can be divided into four pressure regimes: phase **II** (0–0.3 GPa) and three high-pressure (HP) phases, **HP-I** (0.6–2.3 GPa), **HP-II** (3.0–4.8 GPa), and **HP-III** (5.5–8.0 GPa). Accordingly, three pressure-induced transformations are identified at 0.3–0.6 GPa (**II** to **HP-I**), 2.3–3.0 GPa (**HP-I** to **HP-II**), and 4.8–5.5 GPa (**HP-II** to **HP-III**).

The ambient-pressure spectrum already differs slightly from the spectrum recorded at 0.6 GPa, consistent with the **II**→**HP-I** transformation occurring at (0.3–0.6 GPa), similarly to IM_3_PbBr_5_, which exhibited first HP-PT at 0.1–0.7 GPa directly to the ordered LT phase **III**.^29^

Further compression of IM_3_PbCl_5_ leads to the **HP-I**→**HP-II** PT between about 2.3 and 3.0 GPa, evidenced by the appearance of additional Raman bands and clear splitting in the low-wavenumber range (Figure 6b). Comparable, though not universal, splittings are also observed at higher wavenumbers, including the ring stretching region (Figure S9c) and the NH/NH^+^ stretching modes (Figure S10b). These pressure fingerprints resemble several observed on cooling into phase **IV**; however, the correspondence is not uniform for all bands. The splitting pattern, particularly visible for the band at approximately 1196 cm^–1^ (0.3 GPa) into a doublet at 1202 + 1210 cm^–1^ (4.0 GPa), resembles the temperature-driven evolution observed between 240 K (1197 cm^–1^) and 220–230 K (1195 + 1202 cm^–1^), which corresponds to the closely observed PTs at *T*_2_ and *T*_3_. The HP Raman response observed for IM_3_PbBr_5_, together with the stronger HBs of the IM_3_PbCl_5_ structure, suggests that the **HP-I** phase may already be ordered.

Importantly, an additional transformation, **HP-II** to **HP-III**, occurs at higher pressures (4.8–5.5 GPa). In individual peak tracking, this transition is manifested by the emergence of an additional band in the low-wavenumber spectra (Figure 6) and by distinct changes in dω/d*p* for several modes at higher wavenumbers (Table S8). These slope changes are particularly pronounced in the region combining ν_φ_ with δNH and δNH^+^ contributions (Figure S11), indicating a reorganization of the lattice dynamics and hydrogen-bond coupled internal modes upon entering **HP-III**. The transition is captured most clearly by the principal component function (PCF) analysis, which exhibits a distinct anomaly in the 4.8–5.5 GPa range (Figure S12), supporting a genuine structural transformation rather than a gradual spectral drift.

It is noteworthy that similar ordering-associated splitting has been observed for IM_3_PbBr_5_ between 0.1 and 0.7 GPa and between 1.9 and 2.4 GPa,^29^ indicating that analogous spectroscopic changes occur at lower pressures in IM_3_PbBr_5_ than in IM_3_PbCl_5_, where the corresponding transformations occur at higher pressures. Furthermore, the signs of amorphization of the bromide analogue have been observed above 6.0 GPa.^29^ For IM_3_PbCl_5_, the Raman spectrum retains the same spectral features up to 8.0 GPa (Figure 5c). We therefore infer that compression promotes a state with reduced symmetry and increased IM^+^–framework coupling that is closely related to the low-temperature ordered phase; thus, we avoid a definitive identification as the same crystallographic phase based solely on spectroscopy.

Pressure coefficients dω/d*p* estimated for selected Raman bands are collected in Table S8. These values document pressure-induced spectral shifts for several representative modes; however, a direct mode-by-mode comparison remains limited, not only between the bromide and chloride analogues but also between IM_3_PbCl_5_ and IM_2_Pb_3_Cl_8_·0.5H_2_O. This arises from the substantial differences in symmetry, band assignment, and spectral complexity, as well as from mode mixing and pressure-induced changes in the spectral pattern, particularly in IM_3_PbCl_5_. Nevertheless, a clear distinction is observed in the Pb–Cl bending region: IM_2_Pb_3_Cl_8_·0.5H_2_O shows dω/d*p* values of 2.25 and 2.50 cm^–1^ GPa^–1^, whereas the corresponding values for IM_3_PbCl_5_ are substantially larger, reaching 6.30–16.40 cm^–1^ GPa^–1^ depending on the pressure phase.

#### IM_2_Pb_3_Cl_8_·0.5H_2_O

This crystal shows no evidence of a PT in our DSC or XRD measurements. Consistently, neither Raman nor IR spectra collected down to 80 and 7 K, respectively, show any spectral feature characteristic of PTs (Figure 5d,e). Single-crystal X-ray diffraction data indicate that three imidazolium cations in the asymmetric unit are ordered and anchored to the inorganic framework, whereas a fourth interacts with the water molecule and is configurationally disordered. The vibrational response is consistent with these observations through continuous slowing of cation dynamics (librations) on cooling, leading to progressive freezing of molecular motions. In spectroscopic terms, this appears as gradual band narrowing and the suppression of relaxational broadening, without the band splitting or selection-rule changes characteristic of a PT. We attribute this behavior to the relatively large cavity hosting the IM^+^/H_2_O pair and to the three-dimensionally connected Pb–Cl framework, which does not bend as readily as in-chain architectures, thereby permitting kinetic arrest of the organic sublattice without a cooperative lattice instability.

The crystal phase observed above 0.1 GPa remains stable up to at least 4 GPa (Figures 5f and S13). Within this pressure range, the Raman spectra remain well-resolved and do not exhibit changes indicative of a pressure-induced PT. Above 4 GPa, however, the spectral evolution becomes discontinuous, and the spectra are no longer sufficiently consistent for a reliable band tracking and further structural interpretation. For this reason, we did not extend the discussion beyond 4 GPa.

A comparative view of Raman spectra in the CH and NH stretching region for both compounds is presented in Figure S14. It emphasizes the narrower bands in IM_2_Pb_3_Cl_8_·0.5H_2_O, which indicates a more uniform set of N–H and O–H environments, a tighter distribution of hydrogen-bond lengths and angles, and fewer distinct local geometries. In this compound, both the νNH and νCH bands are concentrated within a limited frequency window, consistent with a relatively homogeneous H-bond network. By contrast, IM_3_PbCl_5_ displays a broader νNH spread, reflecting the coexistence of N–H oscillators involved in hydrogen bonds of different strength and geometry, in line with its crystallographic heterogeneity.

### Optical Properties

The optical properties were first studied using RT diffuse reflectance spectroscopy (DRS). The spectra of IM_2_Pb_3_Cl_8_·0.5H_2_O and IM_3_PbCl_5_ exhibit sharp peaks at 298 and 325 nm, respectively, associated with excitonic absorption (Figure S15). The band gap energy (*E*_g_), estimated from the Tauc plot of the absorption edge, is 4.26 eV for IM_2_Pb_3_Cl_8_·0.5H_2_O (Figure S16) and 4.01 eV for IM_3_PbCl_5_ (Figure S17). These values are significantly larger than those reported for other IM^+^-based lead halides, such as IMPbBr_3_ (3.24 eV), IM_2_PbBr_4_ (3.58 eV), and IM_3_PbBr_5_ (3.52 eV).^26^ Similarly, the band gap energies of related 2D hybrids, IMMHyPbBr_4_ and IMMHyPbCl_4_, are lower, amounting to 3.22 and 3.79 eV, respectively.^13^

The increased band gap energies observed for IM_2_Pb_3_Cl_8_·0.5H_2_O and IM_3_PbCl_5_ are consistent with their reduced dimensionality. IM_3_PbCl_5_ has a similar crystal structure to IM_3_PbBr_5_ at RT; thus, it is justified to directly compare their optical outputs. Based on previously reported data for IM_3_PbBr_5_,^26^ the excitonic absorption and band gap were determined to be 378 nm (3.28 eV) and 3.52 eV, respectively. In comparison, IM_3_PbCl_5_ exhibits a blue-shifted excitonic transition and a slightly larger band gap. This difference primarily arises from the substitution of bromide by chloride, which is known to widen the band gap. Higher structural distortions observed for IM_3_PbCl_5_ may further modulate this effect.

Figure 7a presents a direct comparison of normalized photoluminescence (PL) spectra for IM_2_Pb_3_Cl_8_·0.5H_2_O and IM_3_PbCl_5_ registered at 80 K. In both cases, a broad emission extending from about 350 to 900 nm is observed. For IM_3_PbCl_5_, a single band with a maximum at 620 nm and a full width at half-maximum (fwhm) of 182 nm (0.6 eV) is present. In contrast, the emission spectrum of IM_2_Pb_3_Cl_8_·0.5H_2_O is blue-shifted and composed of a dominant band at 594 nm, accompanied by less intense bands at 390, 480, and 560 nm.

**Figure 7:**
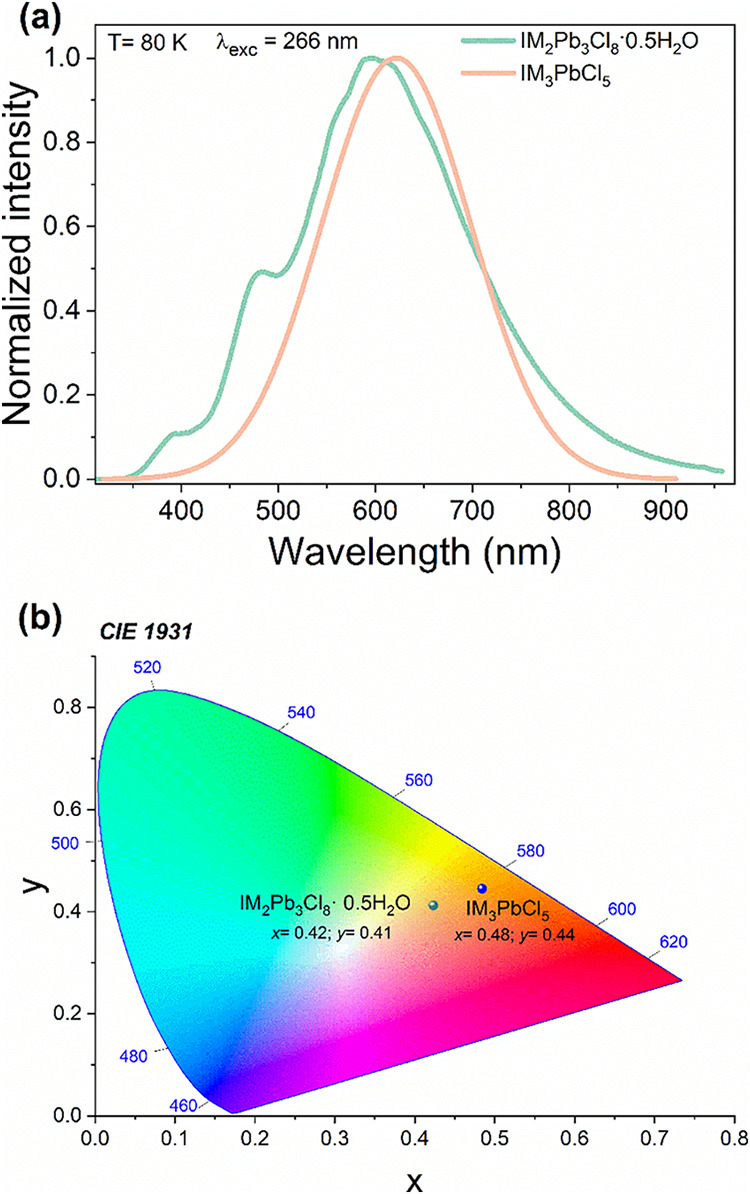
(a) Comparison of normalized PL spectra of IM_2_Pb_3_Cl_8_·0.5H_2_O and IM_3_PbCl_5_ at 80 K and (b) CIE diagrams representing *x*, *y* coordinates of investigated hybrids.

The observed broadband emission is most likely associated with STEs, although contributions from permanent defect states cannot be ruled out. Distinguishing between intrinsic STEs and defect-related emissions is often challenging. Several spectral features, however, support the STE origin of the observed PL, including a large Stokes shift, a broad emission band with substantial fwhm, the absence of a significant temperature-induced shift of maximum, a similar nature of the PL decay curves, and comparable lifetimes. As shown in Figure S18, the emission centered at 590 nm exhibits a slightly longer decay time than that at 480 nm, but their profiles are similar, predominantly suggesting the dominant STE emission mechanism with a still possible contribution from defect states.

Previous studies have demonstrated that low-dimensional hybrid lead halides with corrugated inorganic frameworks can exhibit broadband emission attributed to STE, arising from strong electron–phonon coupling within distorted excited states. The emission bandwidth is often correlated with the degree of structural distortion of the PbX_6_ octahedra.^13,14,26,45,46^ Similarly to other imidazolium-based lead halides, no free-exciton (FE) emission was observed.^13,26^

The IM_3_PbCl_5_ generates the orange-yellow emission at 80 K (Figure 7b), while the LT emission of IM_2_Pb_3_Cl_8_·0.5H_2_O is blue-shifted closer to the center of the color triangle, indicating yellow emission. Due to the low emission intensity of IM_3_PbCl_5_, even at 80 K, the temperature-dependent PL measurements were performed only for IM_2_Pb_3_Cl_8_·0.5H_2_O (Figure 8a). The PL band position and shape remain unchanged over the entire temperature range, indicating the color emission stability. The PL intensity decreases strongly upon heating, with a thermal quenching temperature (T_0.5_) of 155 K (Figure 8b,c). The comparison of the PL spectra of the pristine sample of IM_2_Pb_3_Cl_8_·0.5H_2_O and crystals stored for over a year indicates a good long-term stability (Figure S19).

**Figure 8:**
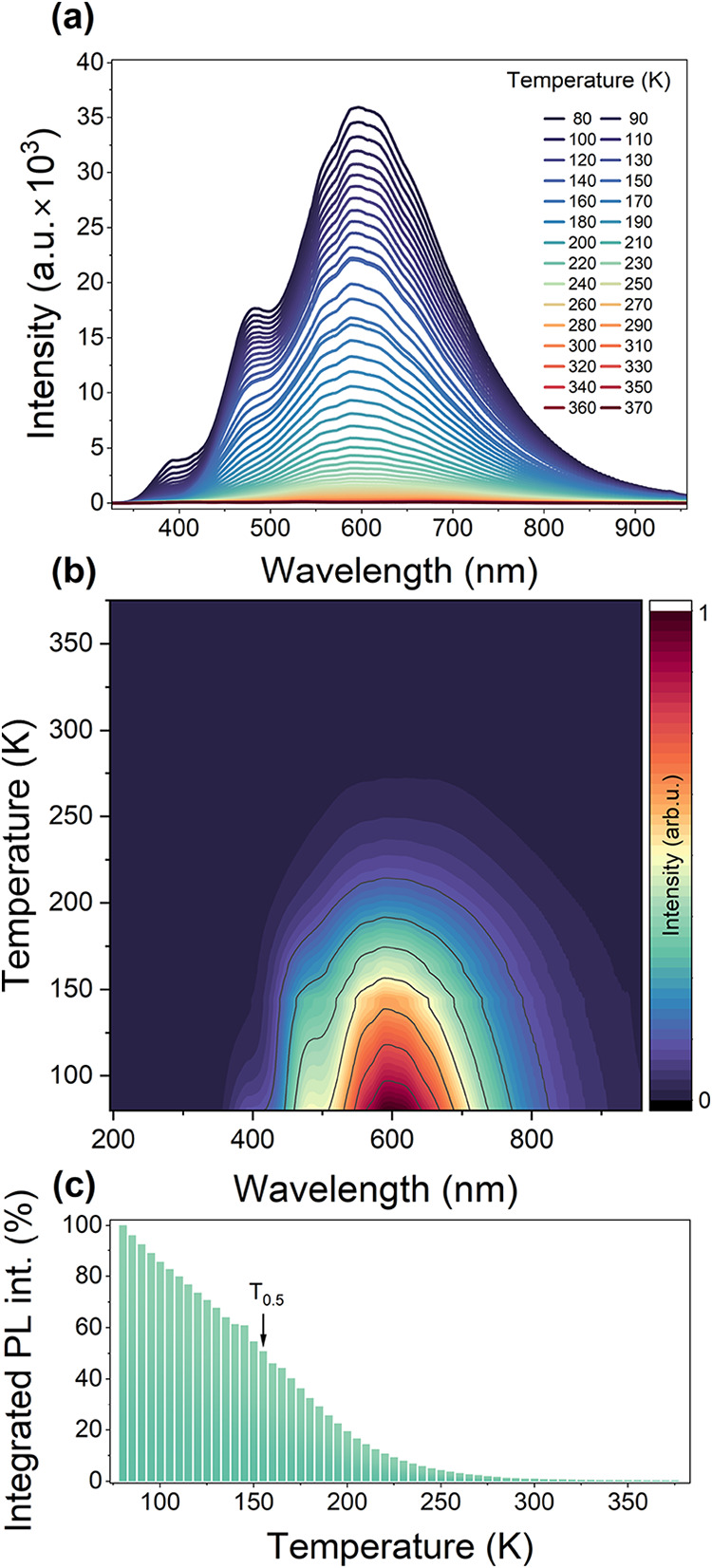
(a) PL spectra measured at various temperatures, (b) PL intensity contour map, and (c) changes of PL intensity with temperature of IM_2_Pb_3_Cl_8_·0.5H_2_O.

## Conclusions

We have synthesized and structurally characterized two new imidazolium lead chlorides, IM_2_Pb_3_Cl_8_·0.5H_2_O and IM_3_PbCl_5_, which extend the family of imidazolium-based hybrid lead halides and show that these materials form a versatile platform for probing how inorganic framework dimensionality and HB control structure, dynamics, and properties. Single-crystal X-ray diffraction demonstrated that IM_3_PbCl_5_ adopts a 1D chain architecture composed of corner-sharing PbCl_6_ units, whereas IM_2_Pb_3_Cl_8_·0.5H_2_O forms a 3D porous framework of edge- and face-sharing PbCl_8_ polyhedra hosting both IM^+^ cations and water molecules. In both structures, N–H···Cl hydrogen bonds provide the primary linkage between the IM^+^ cations and the inorganic scaffold, but the connectivity and topology of the inorganic network impose markedly different constraints on cation ordering and mobility.

Thermal and pressure-dependent studies demonstrated that IM_3_PbCl_5_ is highly prone to ordering phenomena. Upon cooling, the structure evolves from the HT disordered orthorhombic phase **I** (*Cmmm*) to a partially and dynamically disordered monoclinic phase **II** (*P*2/*c*), and to the ordered monoclinic phase **III** (*P*2_1_/*c*), characterized by various octahedral tilting and the formation of additional, more directional N–H···Cl HBs that restrict dynamical IM^+^ movements. The LT phase **IV**, also *P*2_1_/*c*, involves a subtle structural reorganization within the same symmetry, completing the residual thermal librational motions and further enhancing the chain distortions.

Under hydrostatic compression, Raman spectroscopy revealed a sequence of PTs in IM_3_PbCl_5_, from ambient-pressure phase **II** (*P*2/*c*, 0–0.3 GPa) to three HP phases, namely, ordered **HP-I** (0.6–2.3 GPa), **HP-II** (3.0–4.8 GPa), and **HP-III** (above 5.5 GPa). In contrast, IM_2_Pb_3_Cl_8_·0.5H_2_O does not exhibit any temperature- and pressure-induced PTs in the studied thermodynamic conditions (7–420 K and 0–4 GPa).

Dielectric and transport measurements provide a direct picture of the dynamics of ions and molecular dipoles in these materials. In IM_3_PbCl_5_, we detect a clear low-temperature relaxation process in the dielectric spectra: the permittivity and dielectric loss show a pronounced, frequency-dependent peak that shifts to higher temperatures with increasing frequency. The corresponding relaxation times follow an Arrhenius law with an activation energy of about 0.30 eV, which is consistent with thermally activated reorientation of the IM^+^ cations. In IM_2_Pb_3_Cl_8_·0.5H_2_O, no comparable relaxation peak is observed in the studied range, indicating that collective dipolar motions are strongly suppressed. At higher temperatures, both compounds display an increase in AC conductivity with temperature and frequency, characteristic of ionic transport. Analysis of the temperature dependence yields activation energies for long-range ionic conduction of 0.54 and 0.79 eV for IM_3_PbCl_5_ in its high- and intermediate-temperature regimes, and 0.64 eV for IM_2_Pb_3_Cl_8_·0.5H_2_O. Although the microscopic mechanisms differ between the two chlorides, these values show that both materials support mobile ions at elevated temperatures and that the magnitude and temperature dependence of their ionic conductivity can be reliably quantified from the dielectric response.

Optical measurements demonstrated that both compounds have a wide band gap, exhibiting sharp excitonic features at 298 nm for IM_2_Pb_3_Cl_8_·0.5H_2_O and 325 nm for IM_3_PbCl_5_, with corresponding band gaps of 4.26 and 4.01 eV, respectively. At 80 K, both chlorides display broad PL covering the Vis to NIR range of approximately 350–900 nm. IM_3_PbCl_5_ shows a single emission band with a maximum at 620 nm (orange-yellow color) and a large full width at half-maximum of about 0.6 eV. IM_2_Pb_3_Cl_8_·0.5H_2_O exhibits a blue-shifted yellow emission composed of a main band at 594 nm and additional weaker features at 390, 480, and 560 nm. The thermal quenching temperature of IM_2_Pb_3_Cl_8_·0.5H_2_O emission was estimated to be about 155 K, while the band shape remains qualitatively unchanged, indicating that the emission mechanism is preserved in the studied temperature range.

## Supplementary Material



## Notes

Powder X-ray diffraction data, SEM images, DSC traces, dielectric/impedance spectroscopy analysis, and IR/Raman spectra (including selected temperature-/pressure-dependent trends) are provided in the Supporting Information (Figures S1–S19 and Tables S1–S8) (PDF)
