# Supplementary material for: Structural Dynamics of Temperature- and Pressure-Induced Phase Transitions in Hybrid Imidazolium Lead Chlorides
Source: Inorg Chem. 2026 Jun 15;65(25):13827–38. doi: 10.1021/acs.inorgchem.6c00645 (PMC13362192; doi:10.1021/acs.inorgchem.6c00645)
Supplement: Supplementary file 1 [file ic6c00645_si_001.pdf]

Supporting Information

Structural Dynamics of Temperature- and Pressure-Induced Phase Transitions in Hybrid Imidazolium Lead Chlorides

Szymon Smółka, [1,*] Dawid Drozdowski, [1] Dagmara Stefańska, [1] Anna Gągor, [1] Adam Sieradzki, [2] Katarzyna Fedoruk-Piskorska, [2,3] Yuri da Silva Borges, [4] Waldeci Paraguassu, [4] and Maciej Ptak [1]

[1] Institute of Low Temperature and Structure Research, Polish Academy of Sciences, Okólna 2, 50-422 Wrocław, Poland

[2] Faculty of Fundamental Problems of Technology, Wrocław University of Technology, Wybrzeże Wyspiańskiego 27, 50-370 Wrocław, Poland

[3] August Chelkowski Institute of Physics, University of Silesia in Katowice, 41-500 Chorzów, Poland

[4] Department of Physics, Federal University of Pará, Belém, PA, Brazil

corresponding author's email address:
s.smolka@intibs.pl

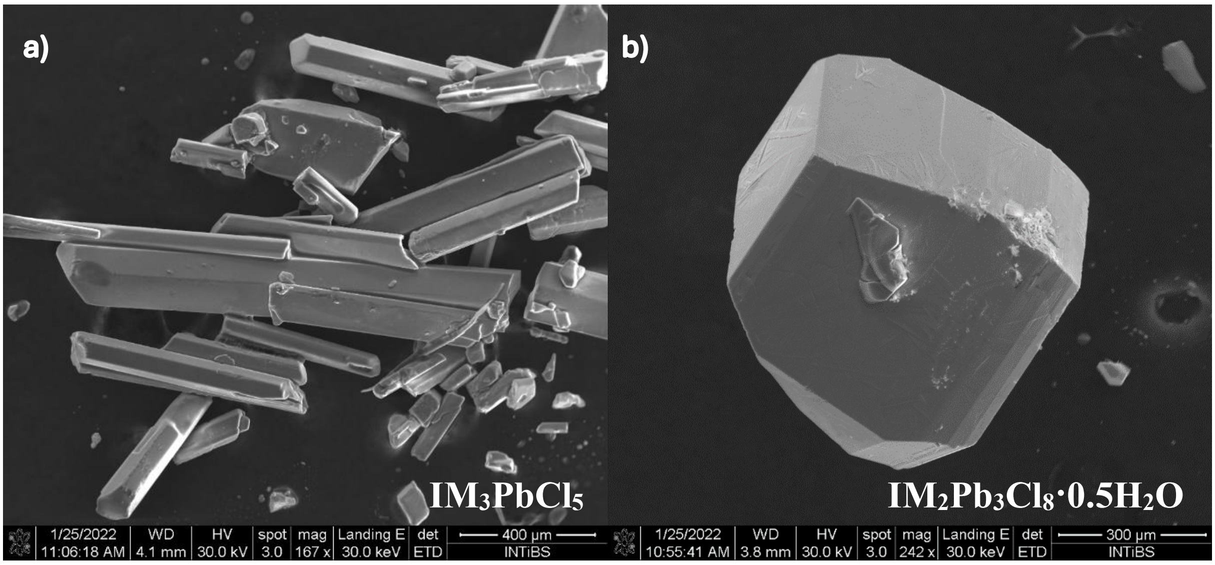

Figure S1. SEM images of the grown (a) IM_3PbCl_5 and (b) $IM_2Pb_3Cl_8 \cdot 0.5H_2O$ crystals.

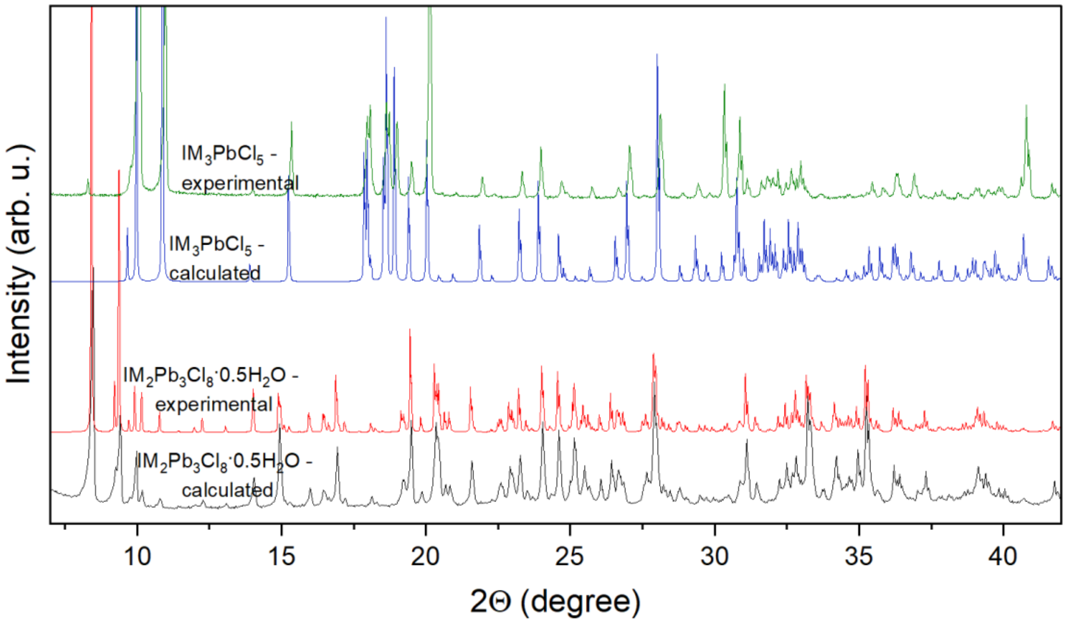

Figure S2. Powder XRD patterns for $IM_2Pb_3Cl_8 \cdot 0.5H_2O$ and IM_3PbCl_5, along with those calculated from the RT single-crystal structures.

Table S1. Experimental and refinement details of IM_3PbCl_5 (M_r = 591.71).

	**Phase I**	**Phase II**	**Phase III**	**Phase IV**
Crystal system, space group	Orthorhombic, *Cmmm*	Monoclinic, *P*2/*c*	Monoclinic, *P*2$_1$/*c*	Monoclinic, *P*2$_1$/*c*
Temperature (K)	405	295	240	120
a, *b*, *c* (Å)	9.109 (3), 18.200 (5), 5.898 (3)	5.812 (3), 9.148 (3), 17.736 (5)	5.775 (3), 17.702 (5), 18.263 (5)	5.747 (3), 17.567 (5), 18.213 (5)
β (°)	90	91.91 (3)	90.07 (3)	90.22 (3)
V (Å^3)	977.7 (7)	942.6 (6)	1867.0 (12)	1838.9 (12)
Z	2	2	4	4
μ (mm^{-1})	9.31	9.66	9.75	9.90
Crystal size (mm)	0.27 × 0.18 × 0.1	0.23 × 0.12 × 0.09		
T_{min}, T_{max}	0.583, 1.000	0.530, 1.000	0.012, 0.033	0.568, 1.000
No. of measured, independent and observed [$I > 2\sigma(I)$] reflections	1580, 605, 531	10321, 1788, 1729	6097, 3100, 2774	21966, 3751, 3623
R_{int}	0.029	0.023	0.025	0.027
(sin $\theta/\lambda)_{max}$ (Å^{-1})	0.625			
$R[F^2 > 2\sigma(F^2)]$, $wR(F^2)$, S	0.036, 0.083, 1.01	0.020, 0.050, 1.20	0.029, 0.063, 1.02	0.016, 0.033, 1.05
No. of reflections	605	1788	3100	3751
No. of parameters	40	94	191	191
$\Delta\rho_{max}$, $\Delta\rho_{min}$ (e Å^{-3})	0.46, -0.87	0.45, -1.37	1.09, -0.93	0.83, -0.53

Table S2. Experimental and refinement details of $IM_2Pb_3Cl_8 \cdot 0.5H_2O$ (M_r = 1052.36).

Crystal system, space group	Monoclinic, *C*2/*c*
Temperature (K)	100
a, *b*, *c* (Å)	21.119 (6), 10.687 (4), 35.625 (9)
β (°)	99.41 (3)
V (Å^3)	7932 (4)
Z	16
μ (mm^{-1})	26.48
Crystal size (mm)	0.25 × 0.14 × 0.08
T_{min}, T_{max}	0.226, 1.000
No. of measured, independent and observed [$I > 2\sigma(I)$] reflections	75637, 7528, 7316
R_{int}	0.041
(sin $\theta/\lambda)_{max}$ (Å^{-1})	0.610
$R[F^2 > 2\sigma(F^2)]$, $wR(F^2)$, S	0.028, 0.075, 1.15
No. of reflections	7528
No. of parameters	303
$\Delta\rho_{max}$, $\Delta\rho_{min}$ (e Å^{-3})	2.85, -3.63

Table S3. Selected geometric parameters of IM_3PbCl_5 (Å, °)

**Phase I, 405 K**			
Pb1—Cl2	2.9489 (15)	Pb1—Cl3^{iii}	2.896 (3)
Pb1—Cl2^{i}	2.9489 (15)	Pb1—Cl3	2.896 (3)
Pb1—Cl3^{ii}	2.896 (3)	Pb1—Cl3^{iv}	2.896 (3)
Cl2—Pb1—Cl2^{i}	180.0	Cl3^{iii}—Pb1—Cl2	90.0
Cl3^{iv}—Pb1—Cl2	90.0	Cl3^{iv}—Pb1—Cl3	180.0
Cl3^{iii}—Pb1—Cl2^{i}	90.0	Cl3^{iv}—Pb1—Cl3^{iii}	88.43 (13)
Cl3—Pb1—Cl2^{i}	90.0	Cl3^{iv}—Pb1—Cl3^{ii}	91.57 (13)
Cl3^{ii}—Pb1—Cl2	90.0	Cl3^{iii}—Pb1—Cl3	91.57 (13)
Cl3^{ii}—Pb1—Cl2^{i}	90.0	Cl3^{iii}—Pb1—Cl3^{ii}	180.0
Cl3—Pb1—Cl2	90.0	Cl3^{ii}—Pb1—Cl3	88.43 (13)
Cl3^{iv}—Pb1—Cl2^{i}	90.0	Pb1^{v}—Cl2—Pb1	180.0
**Phase II, 295 K**			
Pb1—Cl2	2.9063 (15)	Pb1—Cl1	2.8436 (12)
Pb1—Cl2^{vi}	2.9063 (15)	Pb1—Cl3^{vii}	2.9246 (13)
Pb1—Cl1^{vii}	2.8436 (12)	Pb1—Cl3	2.9247 (13)
Cl2^{vi}—Pb1—Cl2	179.43 (5)	Cl1—Pb1—Cl2^{vi}	90.51 (4)
Cl2^{vi}—Pb1—Cl3	88.58 (4)	Cl1—Pb1—Cl1^{vii}	89.41 (5)

Cl2—Pb1—Cl3	91.04 (4)	Cl1^{vii}—Pb1—Cl3^{vii}	176.81 (3)
Cl2—Pb1—Cl3^{vii}	88.58 (4)	Cl1—Pb1—Cl3	176.81 (3)
Cl2^{vi}—Pb1—Cl3^{vii}	91.04 (4)	Cl1—Pb1—Cl3^{vii}	87.53 (4)
Cl1—Pb1—Cl2	89.89 (4)	Cl1^{vii}—Pb1—Cl3	87.53 (4)
Cl1^{vii}—Pb1—Cl2^{vi}	89.89 (4)	Cl3^{vii}—Pb1—Cl3	95.54 (5)
Cl1^{vii}—Pb1—Cl2	90.51 (4)	Pb1—Cl2—Pb1^{viii}	179.43 (5)
**Phase III, 240 K**			
Pb1—Cl1	2.894 (2)	Pb1—Cl2	2.8910 (19)
Pb1—Cl3	2.858 (2)	Pb1—Cl5^{viii}	2.924 (3)
Pb1—Cl4	2.8750 (18)	Pb1—Cl5	2.862 (2)
Cl1—Pb1—Cl5^{viii}	88.49 (6)	Cl4—Pb1—Cl5^{viii}	90.14 (6)
Cl3—Pb1—Cl1	175.39 (6)	Cl2—Pb1—Cl1	85.08 (5)
Cl3—Pb1—Cl4	91.26 (5)	Cl2—Pb1—Cl5^{viii}	93.76 (6)
Cl3—Pb1—Cl2	90.69 (6)	Cl5—Pb1—Cl1	85.50 (6)
Cl3—Pb1—Cl5	92.68 (6)	Cl5—Pb1—Cl4	86.16 (6)
Cl3—Pb1—Cl5^{viii}	93.61 (6)	Cl5—Pb1—Cl2	89.72 (6)
Cl4—Pb1—Cl1	92.83 (6)	Cl5—Pb1—Cl5^{viii}	172.77 (8)
Cl4—Pb1—Cl2	175.52 (6)	Pb1—Cl5—Pb1^{vi}	172.77 (8)
**Phase IV, 120 K**			
Pb1—Cl1	2.9174 (10)	Pb1—Cl2	2.9270 (10)
Pb1—Cl3	2.8399 (10)	Pb1—Cl5^{viii}	2.9406 (18)
Pb1—Cl4	2.8316 (10)	Pb1—Cl5	2.8367 (17)
Cl1—Pb1—Cl2	84.23 (3)	Cl4—Pb1—Cl5	84.95 (3)
Cl1—Pb1—Cl5^{viii}	87.63 (3)	Cl4—Pb1—Cl5^{viii}	88.18 (3)
Cl3—Pb1—Cl1	174.11 (2)	Cl2—Pb1—Cl5^{viii}	96.08 (3)
Cl3—Pb1—Cl2	90.42 (3)	Cl5—Pb1—Cl1	83.32 (3)
Cl3—Pb1—Cl5^{viii}	95.42 (3)	Cl5—Pb1—Cl3	94.26 (3)
Cl4—Pb1—Cl1	93.12 (3)	Cl5—Pb1—Cl2	90.37 (3)
Cl4—Pb1—Cl3	92.01 (3)	Cl5—Pb1—Cl5^{viii}	168.31 (3)
Cl4—Pb1—Cl2	174.87 (2)	Pb1—Cl5—Pb1^{vi}	168.31 (3)

Symmetry codes: (i) x, y, z+1; (ii) -x, y, -z+1; (iii) x, -y, z; (iv) -x, -y, -z+1; (v) x, y, z-1; (vi) x+1, y, z; (vii) -x+1, y, -z+1/2; (viii) x-1, y, z.

Table S4. Selected geometric parameters of $IM_2Pb_3Cl_8\cdot 0.5H_2O$ (Å, °)

Pb3—Cl8	2.8671 (19)	Pb2—Cl9	3.0508 (19)
Pb3—Cl12	2.9748 (18)	Pb2—Cl8	3.0027 (19)
Pb3—Cl11	2.8315 (17)	Pb2—Cl6	2.9751 (19)
Pb3—Cl10	3.018 (2)	Pb2—Cl7	2.8157 (19)
Pb3—Cl10^{i}	2.8973 (19)	Pb2—Cl3^{v}	2.8396 (18)
Pb5—Cl12	2.8417 (19)	Pb2—Cl1^{v}	2.9735 (19)
Pb5—Cl18	3.076 (2)	Pb2—Cl5	3.6754 (12)
Pb5—Cl16	2.7673 (18)	Pb1—Cl4	2.975 (2)
Pb5—Cl2^{ii}	2.9154 (19)	Pb1—Cl2	2.7986 (19)
Pb4—Cl17	3.0770 (19)	Pb1—Cl3	2.8164 (18)
Pb4—Cl19^{iii}	3.0424 (18)	Pb1—Cl1	2.726 (2)
Pb4—Cl4^{iii}	2.8811 (18)	Pb1—Cl5	3.1092 (9)
Pb4—Cl6	2.958 (2)	Cl17—Pb6	2.9540 (18)
Pb4—Cl7	3.086 (2)	Cl19—Pb6	2.8142 (17)
Pb4—Cl13	2.8012 (19)	Cl9—Pb6	2.7832 (18)
Pb4—Cl16^{iv}	2.9052 (19)	Cl18—Pb6	2.6876 (18)
Pb2—Cl17	3.0520 (19)		
Cl8—Pb3—Cl12	79.65 (5)	Cl7—Pb2—Cl17	73.34 (5)
Cl8—Pb3—Cl10	155.08 (5)	Cl7—Pb2—Cl9	142.33 (5)
Cl8—Pb3—Cl10^{i}	95.92 (5)	Cl7—Pb2—Cl8	80.39 (6)
Cl12—Pb3—Cl10	92.71 (5)	Cl7—Pb2—Cl6	81.80 (6)
Cl11—Pb3—Cl8	83.84 (5)	Cl7—Pb2—Cl3^{v}	79.60 (6)
Cl11—Pb3—Cl12	74.99 (5)	Cl7—Pb2—Cl1^{v}	108.38 (6)
Cl11—Pb3—Cl10	71.26 (5)	Cl7—Pb2—Cl5	150.80 (4)
Cl11—Pb3—Cl10^{i}	73.07 (5)	Cl3^{v}—Pb2—Cl17	142.22 (5)
Cl10^{i}—Pb3—Cl12	148.05 (5)	Cl3^{v}—Pb2—Cl9	134.42 (5)
Cl10^{i}—Pb3—Cl10	78.02 (6)	Cl3^{v}—Pb2—Cl8	130.28 (5)
Cl12—Pb5—Cl18	96.20 (5)	Cl3^{v}—Pb2—Cl6	79.67 (5)
Cl12—Pb5—Cl2^{ii}	75.73 (6)	Cl3^{v}—Pb2—Cl1^{v}	71.95 (5)

Cl16—Pb5—Cl12	101.57 (6)	Cl3^{v}—Pb2—Cl5	71.42 (4)
Cl16—Pb5—Cl18	147.68 (5)	Cl1^{v}—Pb2—Cl17	141.70 (5)
Cl16—Pb5—Cl2^{ii}	78.92 (6)	Cl1^{v}—Pb2—Cl9	99.32 (6)
Cl2^{ii}—Pb5—Cl18	79.63 (5)	Cl1^{v}—Pb2—Cl8	72.12 (5)
Cl17—Pb4—Cl7	69.41 (5)	Cl1^{v}—Pb2—Cl6	147.11 (5)
Cl19^{iii}—Pb4—Cl17	134.70 (5)	Cl1^{v}—Pb2—Cl5	66.19 (4)
Cl19^{iii}—Pb4—Cl7	131.48 (5)	Cl4—Pb1—Cl5	92.88 (4)
Cl4^{iii}—Pb4—Cl17	74.75 (5)	Cl2—Pb1—Cl4	86.94 (5)
Cl4^{iii}—Pb4—Cl19^{iii}	72.05 (5)	Cl2—Pb1—Cl3	89.55 (6)
Cl4^{iii}—Pb4—Cl6	84.03 (5)	Cl2—Pb1—Cl5	156.32 (4)
Cl4^{iii}—Pb4—Cl7	143.44 (5)	Cl3—Pb1—Cl4	156.42 (5)
Cl4^{iii}—Pb4—Cl16^{iv}	140.44 (5)	Cl3—Pb1—Cl5	81.20 (4)
Cl6—Pb4—Cl17	70.76 (5)	Cl1—Pb1—Cl4	80.34 (6)
Cl6—Pb4—Cl19^{iii}	75.90 (5)	Cl1—Pb1—Cl2	78.79 (6)
Cl6—Pb4—Cl7	77.70 (5)	Cl1—Pb1—Cl3	76.10 (6)
Cl13—Pb4—Cl17	72.37 (5)	Cl1—Pb1—Cl5	77.84 (5)
Cl13—Pb4—Cl19^{iii}	137.76 (5)	Pb2—Cl17—Pb4	88.73 (4)
Cl13—Pb4—Cl4^{iii}	91.94 (5)	Pb6—Cl17—Pb4	100.86 (5)
Cl13—Pb4—Cl6	142.66 (5)	Pb6—Cl17—Pb2	91.30 (5)
Cl13—Pb4—Cl7	84.16 (5)	Pb6—Cl19—Pb4^{iii}	93.89 (5)
Cl13—Pb4—Cl16^{iv}	98.82 (5)	Pb6—Cl9—Pb2	94.72 (5)
Cl16^{iv}—Pb4—Cl17	144.75 (5)	Pb4^{iii}—Cl4—Pb1	97.77 (5)
Cl16^{iv}—Pb4—Cl19^{iii}	74.57 (5)	Pb3—Cl8—Pb2	96.28 (6)
Cl16^{iv}—Pb4—Cl6	107.80 (5)	Pb5—Cl12—Pb3	97.89 (5)
Cl16^{iv}—Pb4—Cl7	75.84 (5)	Pb4—Cl6—Pb2	92.49 (5)
Cl17—Pb2—Cl5	129.73 (4)	Pb3—Cl11—Pb3^{i}	95.31 (8)
Cl9—Pb2—Cl17	69.10 (5)	Pb2—Cl7—Pb4	93.01 (5)
Cl9—Pb2—Cl5	64.51 (4)	Pb3^{i}—Cl10—Pb3	90.06 (5)
Cl8—Pb2—Cl17	70.53 (5)	Pb6—Cl18—Pb5	93.60 (5)
Cl8—Pb2—Cl9	84.53 (5)	Pb5—Cl16—Pb4^{vi}	101.97 (6)
Cl8—Pb2—Cl5	121.41 (4)	Pb1—Cl2—Pb5^{ii}	104.82 (6)
Cl6—Pb2—Cl17	70.89 (5)	Pb1—Cl3—Pb2^{v}	98.55 (6)
Cl6—Pb2—Cl9	88.67 (6)	Pb1—Cl1—Pb2^{v}	97.45 (6)
Cl6—Pb2—Cl8	140.70 (5)	Pb1—Cl5—Pb2	102.13 (2)
Cl6—Pb2—Cl5	89.46 (4)		

Symmetry codes: (i) -*x*+1, *y*, -*z*+1/2; (ii) -*x*+1, -*y*+2, -*z*+1; (iii) -*x*+1, -*y*+1, -*z*+1; (iv) *x*, *y*-1, *z*; (v) -*x*+3/2, -*y*+3/2, -*z*+1; (vi) *x*, *y*+1, *z*.

Table S5. Selected hydrogen-bond parameters of IM$_3$PbCl$_5$.

D*—H···*A	***D*—H (Å)**	**H···*A* (Å)**	***D*···*A* (Å)**	***D*—H···*A* (°)**
**Phase II, 295 K**				
N3—H3···Cl3^{i}	0.86	2.73	3.333 (5)	128.5
N3—H3···Cl3^{ii}	0.86	2.90	3.458 (5)	124.0
N1—H1···Cl1	0.86	2.36	3.193 (5)	162.9
**Phase III, 240 K**				
N6—H6···Cl5	0.87	2.64	3.394 (9)	145.3
N1—H1···Cl4	0.87	2.58	3.273 (7)	137.7
N11—H11···Cl2	0.87	2.60	3.264 (8)	133.6
**Phase IV, 120 K**				
N6—H6···Cl3	0.88	2.78	3.353 (3)	123.8
N6—H6···Cl7	0.88	2.54	3.290 (4)	144.1
N8—H8···Cl6^{iii}	0.88	2.32	3.154 (3)	159.2
N1—H1···Cl5	0.88	2.46	3.193 (3)	141.5
N1—H1···Cl5^{iv}	0.88	2.78	3.327 (3)	121.3
N3—H3···Cl4^{v}	0.88	2.29	3.161 (3)	169.9
N13—H13···Cl3^{vi}	0.88	2.28	3.147 (3)	167.6
N11—H11···Cl6	0.88	2.55	3.209 (3)	132.0

Symmetry codes: (i) -*x*, *y*, -*z*+1/2; (ii) *x*-1, -*y*, *z*+1/2; (iii) *x*+1, -*y*+1/2, *z*+1/2; (iv) -*x*+1, -*y*, -*z*+1; (v) -*x*+2, -*y*, -*z*+1; (vi) *x*+1, *y*, *z*.

Table S6. Selected hydrogen-bond parameters of $IM_2Pb_3Cl_8\cdot 0.5H_2O$.

D*—H···*A	***D*—H (Å)**	**H···*A* (Å)**	***D*···*A* (Å)**	***D*—H···*A* (°)**
N16—H16···Cl10	0.88	2.44	3.197 (7)	144.3
N16—H16···Cl13	0.88	2.59	3.198 (7)	126.5
N1—H1···Cl1	0.88	2.44	3.104 (7)	132.6
N6—H6···Cl9^{i}	0.88	2.67	3.265 (7)	126.3
N6—H6···Cl5	0.88	2.47	3.216 (7)	143.1
N8—H8···Cl6^{ii}	0.88	2.55	3.331 (7)	148.3
N8—H8···Cl3^{iii}	0.88	2.75	3.268 (7)	119.3
N3—H3···O1^{iv}	0.88	2.35	3.059 (12)	138.3
N11—H11···O1	0.88	2.30	3.074 (12)	146.2
N18—H18···Cl12^{iii}	0.88	2.47	3.267 (7)	150.9
N18—H18···Cl11^{iii}	0.88	2.66	3.166 (7)	117.6
C14A—H14A···O1^{v}	0.95	2.17	3.01 (2)	147.2

Symmetry codes: (i) -*x*+3/2, -*y*+3/2, -*z*+1; (ii) -*x*+3/2, -*y*+1/2, -*z*+1; (iii) *x*, *y*-1, *z*; (iv) *x*+1/2, -*y*+1/2, *z*+1/2; (v) -*x*+1/2, *y*-1/2, -*z*+1/2.

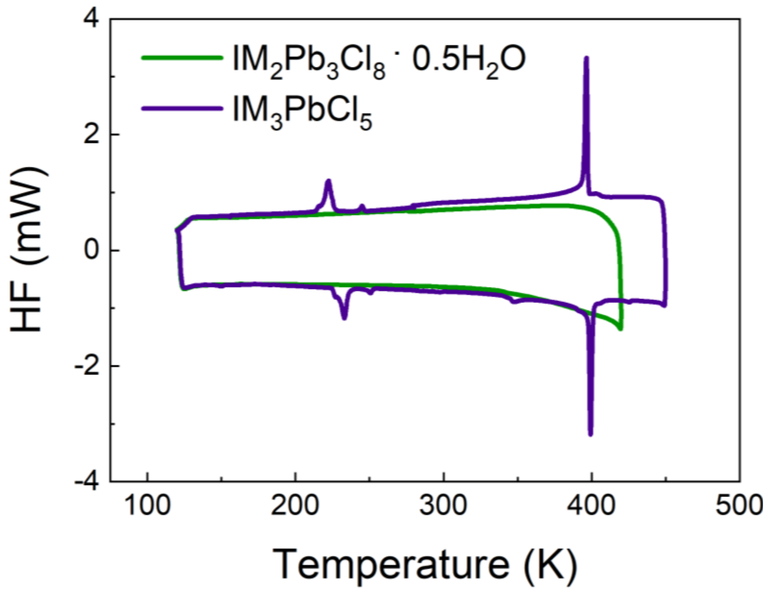

Figure S3. DSC trace for IM_3PbCl_5 (purple) and $IM_2Pb_3Cl_8\cdot 0.5H_2O$ (green).

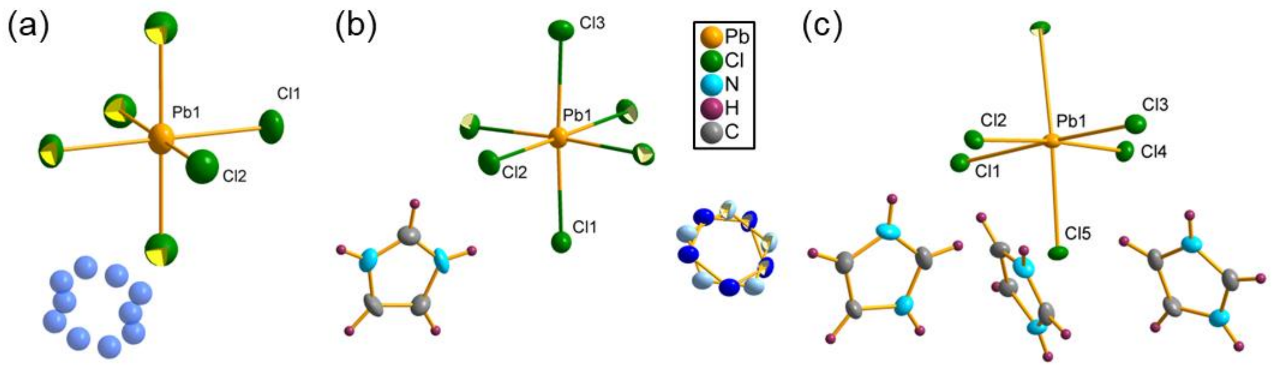

Figure S4. Basic building units of IM_3PbCl_5 at (a) 405 K (orthorhombic, *Cmmm*), (b) 295 K (monoclinic, *P*2/*c*), and (c) 100 K (monoclinic, $P2_1/c$). Symmetry-generated atoms are represented as octants.

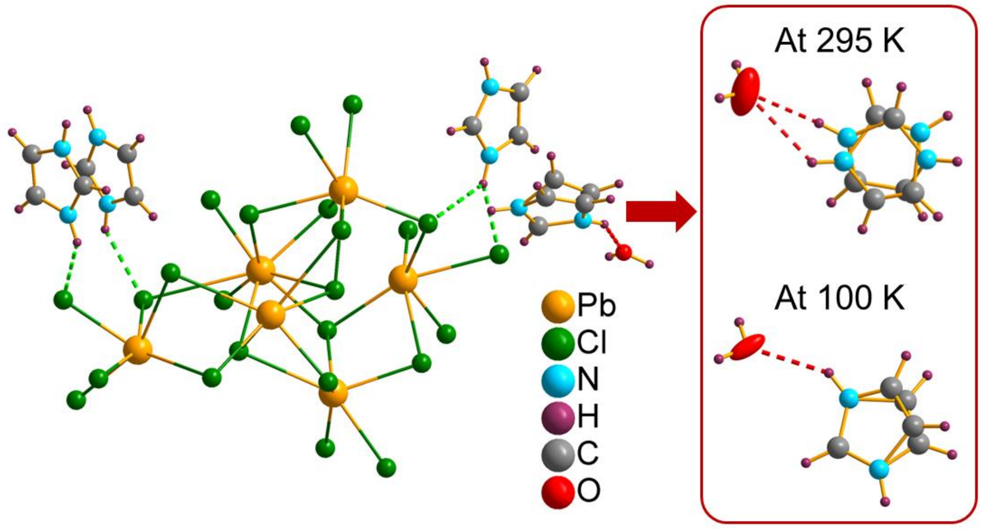

Figure S5. Basic building units of $IM_2Pb_3Cl_8 \cdot 0.5H_2O$ at 100 K (monoclinic, *C*2/*c*). Green (red) dashed lines denote N–H···Cl (N–H···O) HBs. Symmetry-generated atoms are represented as octants.

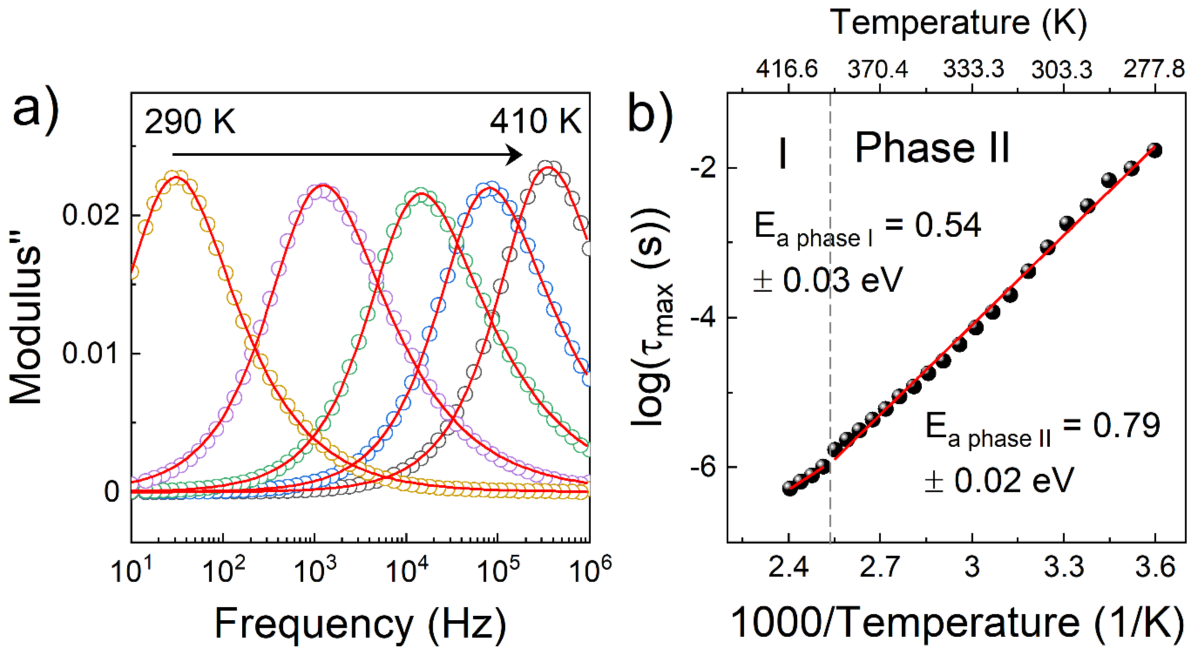

Figure S6. (a) The Havriliak-Negami function (red curve) fitted to the imaginary part of the modulus for IM_3PbCl_5 and (b) the dependence of the logarithm of relaxation time log(τ_{max}) on the inverse temperature (1000/*T*) is fitted using the Arrhenius equation.

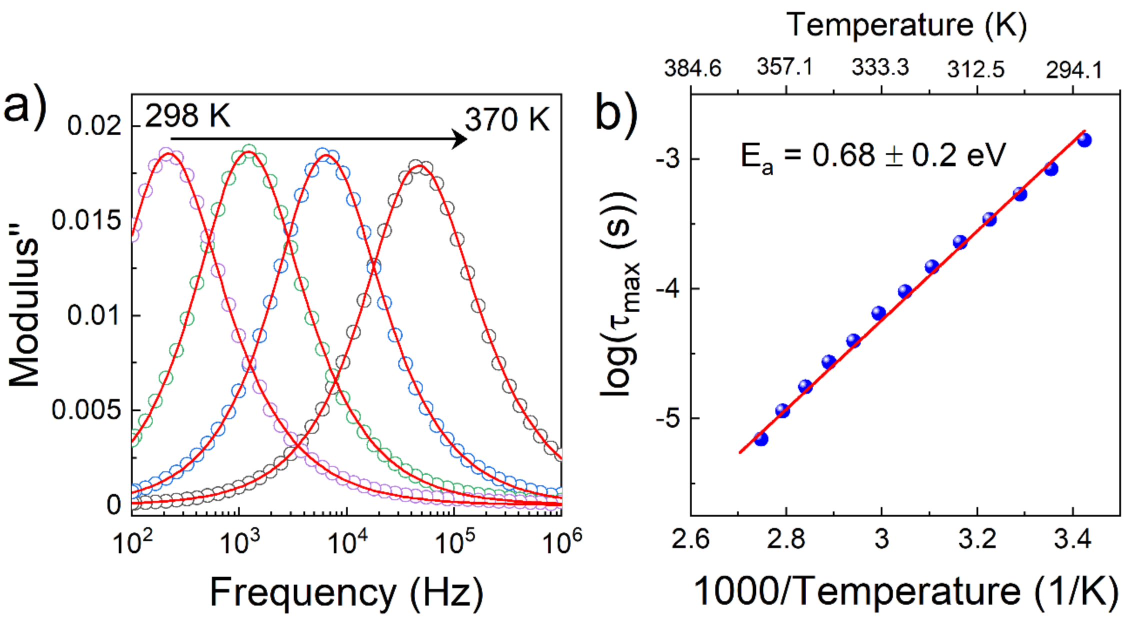

Figure S7. (a) Fitting of the imaginary part of the electric modulus for $IM_2Pb_3Cl_8{\cdot}0.5H_2O$ using the Havriliak-Negami relaxation function (red curve) and (b) temperature dependence of the relaxation time log(τ_{max}) presented as a function of inverse temperature (1000/*T*).

Table S7. Raman and IR bands of IM_3PbCl_5 and $IM_2Pb_3Cl_8{\cdot}0.5H_2O$ together with their relative intensities and band assignments.

**IM_3PbCl_5**		**$IM_2Pb_3Cl_8{\cdot}0.5H_2O$**		**Assignment**
**Raman (80K)**	**IR (7K)**	**Raman (80K)**	**IR (7K)**	
3243_{sh}, 3273_{w}	$3256_{b,m}$, $3272_{b,m}$, 3292_{sh}, 3315_{sh}	3261_{vw}, 3286_{vw}	3287_{sh}, 3308_{w},	νNH + νNH$^+$
3102_{w}, 3119_{m}, 3131_{s}, 3138_{w}, 3144_{w}, 3156_{w}, 3170_{m}, 3177_{s}	3104_{m}, 3115_{m}, 3129_{m}, 3135_{m}, 3143_{sh}, 3154_{m}, 3168_{w}, 3175_{sh}, $3205_{b,m}$	3120_{w}, 3132_{vs}, 3138_{s}, 3156_{m}, 3166_{m}	3099_{sh}, 3115_{m}, 3126_{m}, 3135_{m}, 3153_{m}, 3162_{m}, 3210_{vs}, 3237_{s}, 3254_{m}, 3276_{m}	νCH
2978w, 3001_{w}, 3036_{vw}, 3060b	2998_{m}, 3020_{m}, 3045_{m},	3025_{vw}, 3036_{vw}	3009_{sh}, 3022_{sh}, 3027_{w}, 3037_{w}	νNH + νNH$^+$
2859_{vw}, 2885_{vw}	$1600\text{-}2800_{vw}$		$1600\text{-}2800_{vw}$	overtones
1582_{vw}	1568_{sh}, 1578_{s}	1567_{vw}, 1575_{vw}	1568_{vs} 1575_{vs}, 1587_{w}	ν_ϕ
1520_{vw}, 1531_{vw},	1520_{sh}, 1527_{w},		1520_{sh}, 1528_{w}	δCH + ν_ϕ
1419_{w}, 1436_{vw}, 1444_{vw}, 1454_{w}	1418_{s}, 1422_{m}, 1435_{sh},1443_{sh}	1406_{w}, 1418_{vw}, 1428_{w}, 1438_{vs}, 1441_{vs}	1400_{m}, 1433_{s}, 1440_{sh} 1455sh	ν_ϕ + δNH + δNH$^+$
1237_{vw}, 1252_{vw}	1236_{vw}, 1252_{vw}, 1298_{w}	1252_{vw}, 1291_{vw}, 1305_{vw}	1246_{vw}, 1250_{vw}, 1290_{vw}, 1300_{vw}	δCH
1195_{vs}, 1206_{vs}	1172_{w}, 1186_{vw}, 1195_{w}, 1207_{w}	1182_{m}, 1189_{s}, 1194_{sh}, 1200_{vw}	1182_{m}, 1200_{vw},	ν_ϕ
1117_{w}, 1163_{m}	1116_{vw}, 1121_{vw}, 1159_{m}	1112_{m}, 1123_{vw}, 1142_{sh}, 1147_{w}, 1159_{w} 1167_{sh}	1108_{vw}, 1121_{vw}, 1154_{vw}, 1161_{vw}	δCH + ν_ϕ
1081_{m}, 1087_{m}, 1100_{vw}, 1106_{vw}	1079_{s}, 1085_{m}, 1100_{w}, 1105_{vw}	1097_{vw}, 1106_{vw}	1071_{s}, 1079_{m}, 1095_{vw}	ν_ϕ + δNH + δNH$^+$
1041_{vw}, 1052_{w}	1038_{s}, 1048_{s}	1040_{vw}, 1049_{vw}	1029_{m}, 1040_{s}, 1047_{s}	ν_ϕ + δCH
903_{vw}, 915_{vw}, 925_{vw}	904_{sh}, 912_{sh}, 918_{w}, 925_{vw}	906_{vw}, 919_{vw}, 926_{vw}	872_{vw}, 892_{vw}, 904_{vw}, 914_{sh}, 917_{vw}, 941_{vw}	δ_ϕ

810_{vw}, 827_{vw} 804_{vw}, 892_{vw}	815_{s}, 828_{s}, 883_{w}, 897_{m}	884_{vw}, 895_{vw}	806_{w}, 812_{w}, 821_{w}, 830_{vw}, 840_{vw}, 845_{vw},	γCH
760_{vw}, 764_{sh}, 776_{vw}	741_{m}, 760_{vs}, 771_{vs}, 778_{vs}, 781_{vs}	758_{vw}, 769_{vw}, 776_{vw}, 791_{vw}	739_{m}, 764_{sh}, 768_{s}, 772_{s}, 783_{sh}, 792_{m}	$\gamma NH + \gamma NH^+$
450_{vw}, 619_{vw}, 629_{vw}	617_{s}, 625_{vs}	626_{vw}	515_{vw}, 557_{vw}, 580_{vw}, 597_{vw}, 623_{s}	γ_{ϕ}
160_{vw}, 170_{vw}, 195_{vw}		165_{m}, 181_{m}, 206_{w}, 227_{s}		νPbCl
117_{m}, 131_{m}		119_{s}, 135_{sh}		νPbCl + T’
93_{s}, 104_{m}		80_{s}, 103_{vs}		δPbCl + L
59_{w}, 68_{m}, 75_{w}, 82_{w}		57_{sh}, 65_{m}		δPbCl + T’ + L

Key: b, broad; vs, very strong; s, strong; m, medium; w, weak; vw, very weak; ν, stretching; ν_{ϕ}, ring stretching; δ, in-plane bending; δ_{ϕ}, in-plane ring bending; γ, out-of-plane bending; γ_{ϕ}, out-of-plane ring bending; T’, translation; L, libration.

Table S8. Linear fitting results of pressure-dependencies of Raman bands of $IM_2Pb_3Cl_8\cdot0.5H_2O$ and IM_3PbCl_5 for selected bonds.

**$IM_2Pb_3Cl_8\cdot0.5H_20$**		**IM_3PbCl_5 phase HP-I**		**IM_3PbCl_5 phase HP-II**		**IM_3PbCl_5 phase HP-III**		**Assignment**
**ω_0 (cm^{-1})**	**$d\omega/dp$ ($cm^{-1}GPa^{-1}$)**	**ω_0 (cm^{-1})**	**$d\omega/dp$ ($cm^{-1}GPa^{-1}$)**	**ω_0 (cm^{-1})**	**$d\omega/dp$ ($cm^{-1}GPa^{-1}$)**	**ω_0 (cm^{-1})**	**$d\omega/dp$ ($cm^{-1}GPa^{-1}$)**	
3165.7	7.88	3173.8	8.13	3172.1	7.93	3186.2	5.83	νCH
3153.1	3.56			3170.0	8.17	3186.9	5.15	
1443	2.48	1446.1	1.15	1438.9	3.63	1446.4	2.36	ν_{φ} + δNH + δNH^+
				1438.9	1.90	1439.3	1.47	
		1418.9	1.25	1418.9	1.66	1420.2	1.57	
				1413.1	1.58	1422.1	0.02	
1188.9	1.63	1194.9	2.57	1198.8	3.05	1202.5	2.57	ν_{φ}
				1196.4	1.64	1198.2	1.18	
1106.5	2.48	1048.5	0.66	1044.0	2.25	1044.1	2.28	ν_{φ} + δCH
				1044.5	0.87	1045.6	0.74	
224.2	6.62			176.4	10.28	220.7	9.99	νPbCl
		185.5	8.02			194.2	10.13	
175	10.47			161.9	21.21	184.4	7.36	
64.4	2.5	92.9	16.40	115.8	6.30	118.1	10.14	δPbCl
42.1	2.25			108.4	11.63	110.8	7.68	

Key: ν, stretching; ν_{ϕ}, ring stretching; δ, in-plane bending

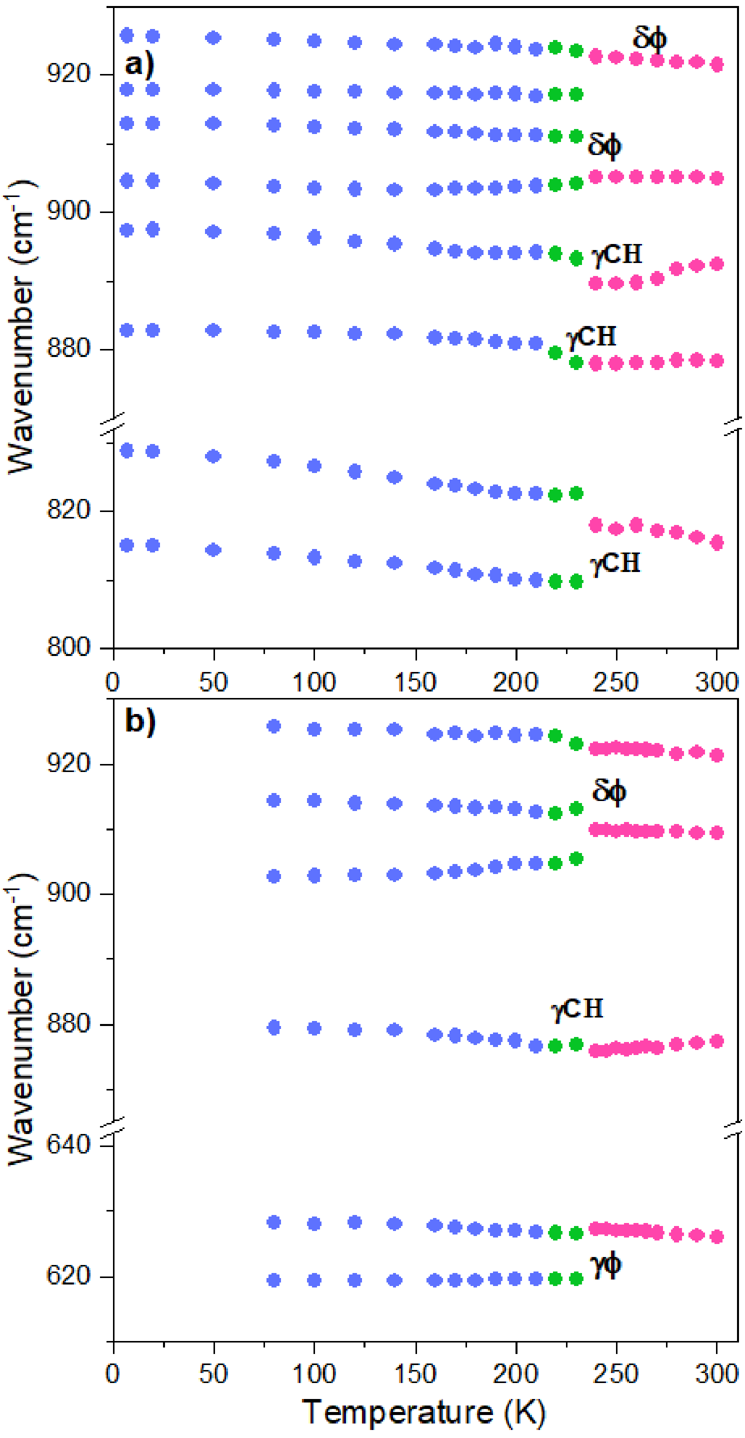

Figure S8. Temperature evolution of positions of selected IR (a) and Raman (b) bands of IM_3PbCl_5. Colors of full circles corresponds to different phase (pink – phase **II**, green phase **III**, blue phase **IV**). Each of the presented modes has labels with assignments: δ_ϕ, in-plane ring bending; γ, out-of-plane bending; γ_ϕ, out-of-plane ring bending.

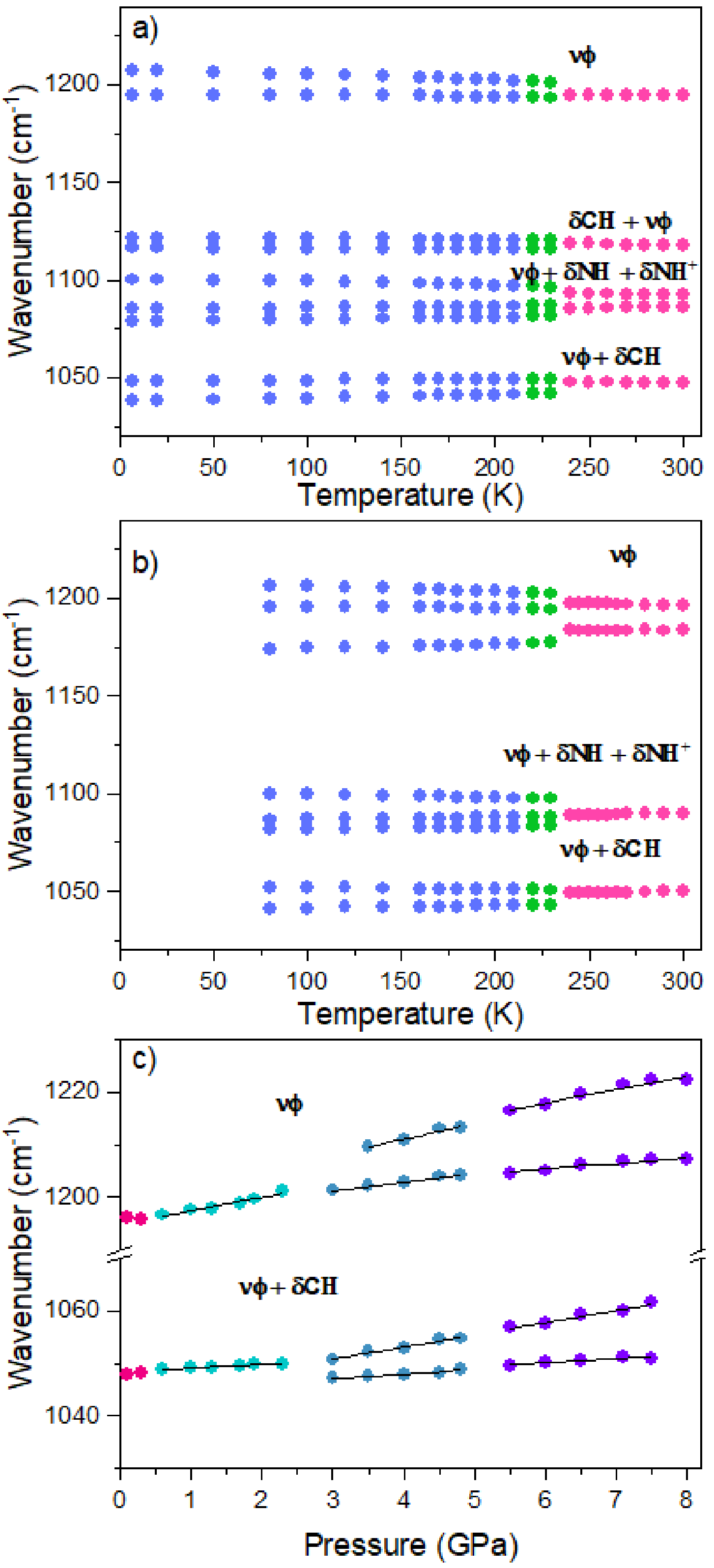

Figure S9. Temperature evolution of positions of selected IR (a) and Raman (b) bands of IM_3PbCl_5. Colors of full circles correspond to different phases (pink – phase **II**, green phase **III**, blue phase **IV**). Pressure-dependent evolution for the same region is presented in (c) (red **II**, cyan **HP-I**, dark cyan **HP-II**, purple **HP-III**). Each of the presented modes has labels with assignments: ν_ϕ, ring stretching; δ, in-plane bending.

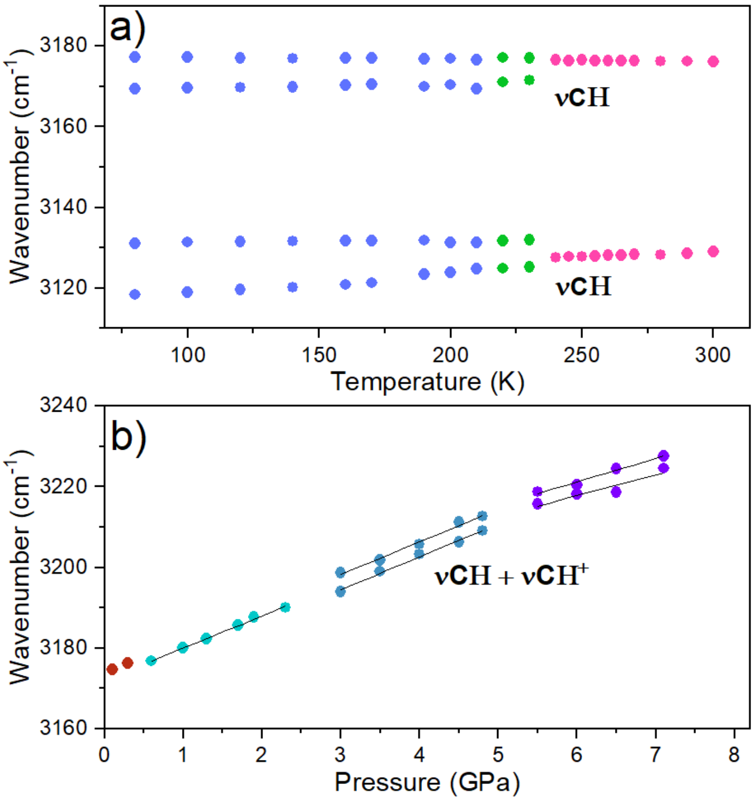

Figure S10. Evolution of positions of selected (a) temperature- and (b) pressure-dependent Raman stretching νCH modes of IM_3PbCl_5. Colors of full correspond to different temperature phases (pink – phase **II**, green phase **III**, blue phase **IV**) and pressure phases (red **II**, cyan **HP-I**, dark cyan **HP-II**, purple **HP-III**).

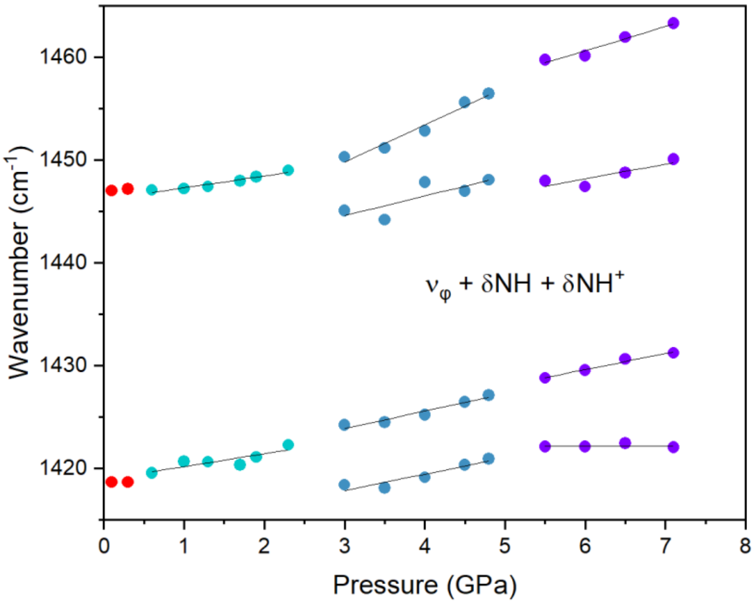

Figure S11. Evolution of positions of selected pressure-dependent Raman bands of IM_3PbCl_5. Colors of full circles correspond to different pressure phases (red **II**, cyan **HP-I**, dark cyan **HP-II**, purple **HP-III**). Each of the presented modes has labels with assignments: ν, stretching; δ, in-plane bending.

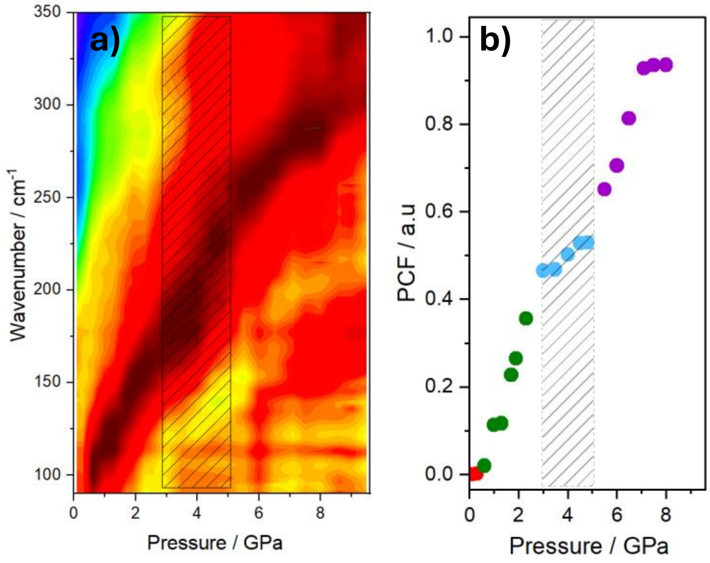

Figure S12. Pressure-dependent Raman response of IM_3PbCl_5 in the low-wavenumber region. (a) Raman intensity map (wavenumber vs pressure) showing pressure-driven reorganization of lattice modes; the hatched area indicates phase **HP-II**. (b) Normalized principal component function (PCF) calculated from the spectra in panel (a) as a function of pressure, exhibiting an anomaly in the hatched region consistent with phase transition pressure regimes.

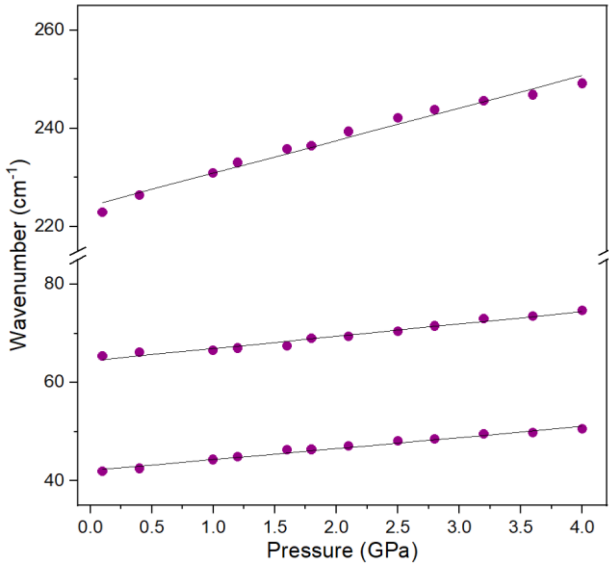

Figure S13. Evolution of positions of selected pressure-dependent Raman bands of $IM_2Pb_3Cl_8 \cdot 0.5H_2O$ in the low-wavenumber region.

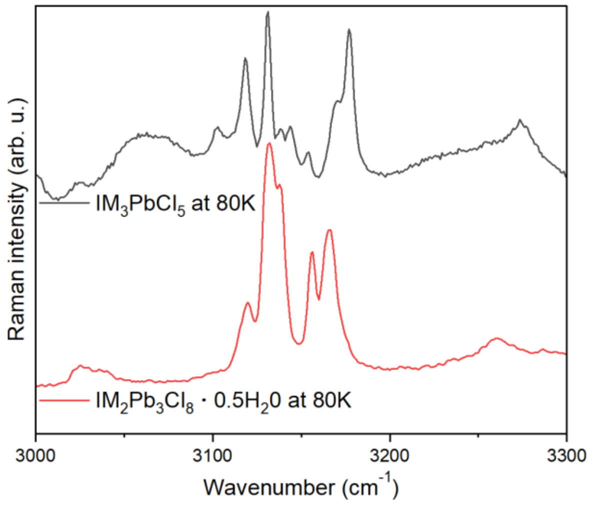

Figure S14. Raman spectra in the CH and NH stretching region collected at 80 K for $IM_2Pb_3Cl_8 \cdot 0.5H_2O$ and IM_3PbCl_5.

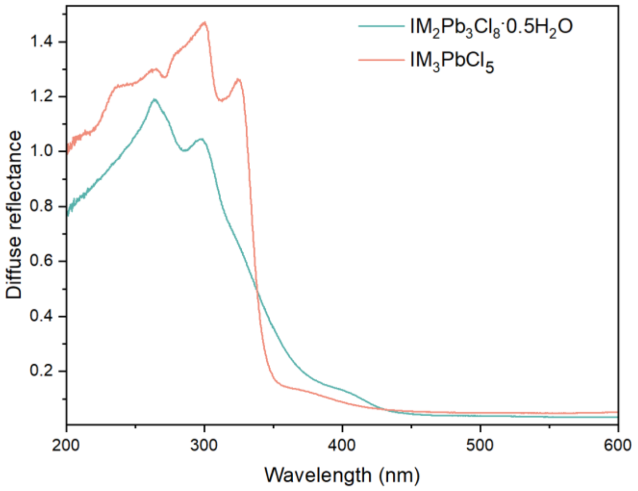

Figure S15. 300 K DRS spectra of $IM_2Pb_3Cl_8 \cdot 0.5H_2O$ and IM_3PbCl_5.

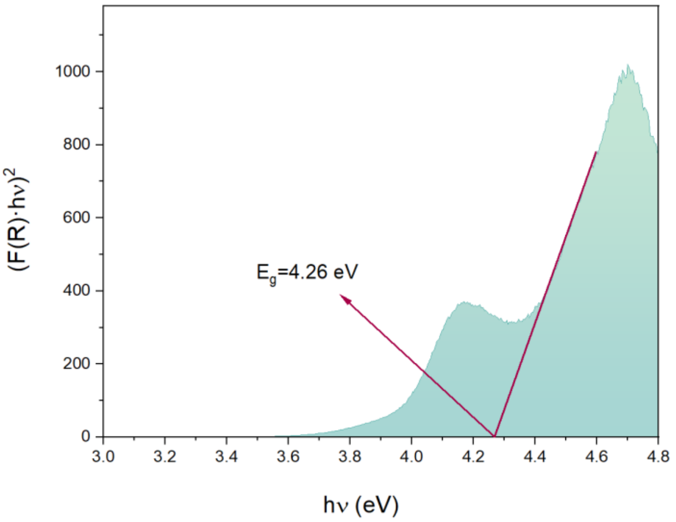

Figure S16. Energy band gap of $IM_2Pb_3Cl_8 \cdot 0.5H_2O$ estimated by *x*-axis intersection points of the linear fits of the Tauc plots.

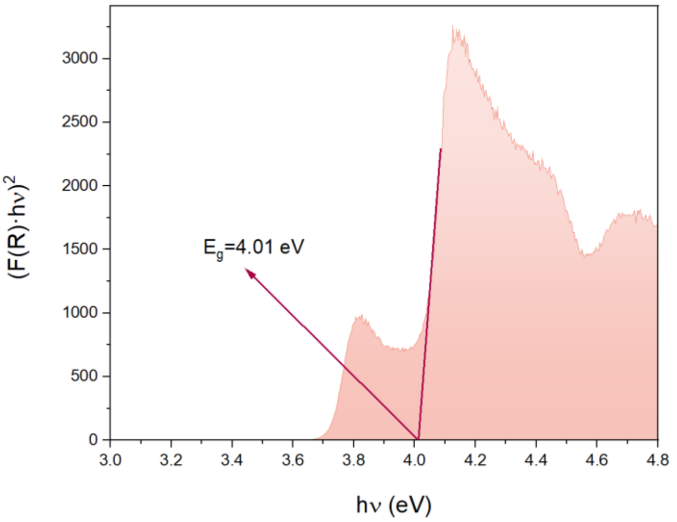

Figure S17. Energy band gap of IM_3PbCl_5 estimated by *x*-axis intersection points of the linear fits of the Tauc plots.

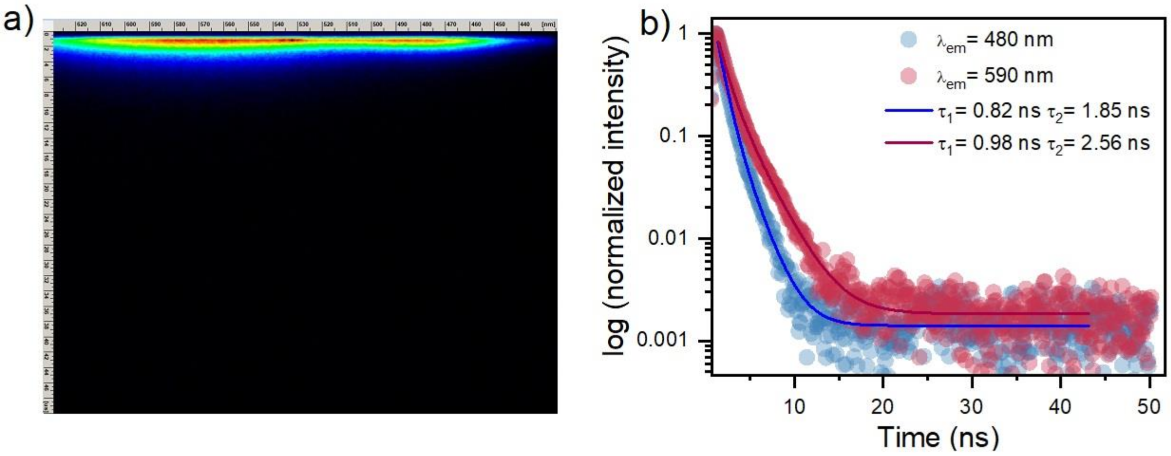

Figure S18. Time resolved PL of $IM_2Pb_3Cl_8{\cdot}0.5H_2O$ recorded at 80 K and 266 nm excitation (a) and PL decay curves (b).

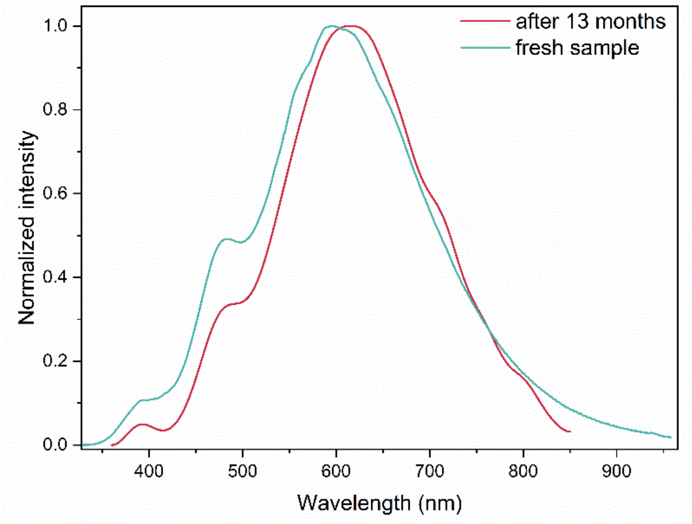

Figure S19. Collation of PL (λexc= 266 nm) of $IM_2Pb_3Cl_8{\cdot}0.5H_2O$ sample immediately after synthesis and after 13 months stored in an air atmosphere.
